# CD8^+^ effector memory and regulatory T cell dynamics predict subclinical atherosclerotic plaque formation in healthy aging

**DOI:** 10.1186/s12979-026-00588-2

**Published:** 2026-08-15

**Authors:** Christopher Weyh, Vincent Größer, Luciele Guerra Minuzzi, Torsten Frech, Kristina Gebhardt, Svenja Nolte, Theresa Regine Dombrowski, Tim Böttrich, Manuela Andrea Hoffmann, Natascha Sommer, Robert Ringseis, Klaus Eder, Samuel Sossalla, Pascal Bauer, Karsten Krüger

**Affiliations:** 1 Department of Exercise Physiology and Sports Therapy, Institute of Sports Science, Justus- Liebig-University, Kugelberg 62, Giessen, 35394 Germany; 2 Institute for Preventive Medicine of the German Armed Forces, Andernach, 56626 Germany; 3 Department of Cardiology and Angiology, Excellence Cluster Cardio-Pulmonary Institute (CPI), Justus-Liebig-University Giessen, Giessen, 35392 Germany; 4 Faculty of Sport Sciences and Physical Education, CIPER, University of Coimbra, Coimbra, 3040-248 Portugal; 5 Department of Nuclear Medicine, University Medical Center of the Johannes Gutenberg- University, Mainz, 55131 Germany; 6 Department of Internal Medicine, Universities of Giessen and Marburg Lung Center (UGMLC), Member of the German Center for Lung Research (DZL), Excellence Cluster Cardio- Pulmonary Institute (CPI), Justus-Liebig University, Giessen, 35394 Germany; 7 Institute of Animal Nutrition and Nutrition Physiology, Justus Liebig University Giessen, Giessen, 35390 Germany; 8 Center for Sustainable Food Systems, Justus Liebig University Giessen, Senkenbergstraße 3, Giessen, 35390 Germany; 9 Department of Cardiology, Kerkhoff Clinic GmbH, Bad Nauheim, Germany

**Keywords:** Subclinical atherosclerosis, T cell immunosenescence, Effector regulatory imbalance, Vascular aging

## Abstract

**Background:**

Subclinical atherosclerotic plaque (SAP) develops silently in older adults. Biological changes that predict plaque formation are not well defined. We examined whether circulating immunological, metabolic, and functional measures identify vascular vulnerability before clinical disease.

**Methods:**

In a three-year longitudinal study, 49 healthy older adults (63.8 ± 3.8 years) underwent carotid ultrasound, T cell phenotyping, serum protein profiling, body composition assessment, and fitness testing at baseline and follow-up. At follow-up, participants were classified as no SAP, new SAP or persistent SAP. We analyzed baseline determinants and within-person changes to predict incident plaque formation.

**Results:**

New SAP occurred in 30.3% of those initially plaque-free. At baseline, lower frequencies of CD8^+^ effector memory (EM) T cells and higher frequencies of CD8^+^ effector memory re-expressing CD45RA (EMRA) T cells and regulatory T cells (Tregs) were associated with higher odds of new SAP. Over time, expansion of CD8^+^ EM T cells was the most consistently associated independent variable of new SAP, accompanied by declines in Tregs and in the Treg/Teff ratio. Vascular cell adhesion molecule-1 (VCAM-1) at baseline was an additional independent predictor. Increases in visceral fat and declines in VO_2_peak were linked to new SAP, but immune markers were more robust than metabolic variables or serum cytokines.

**Conclusion:**

Adaptive immune remodeling follows a phase-dependent pattern in which CD8^+^ EM reductions and later CD8^+^ EM expansion with Treg decline signal emerging vascular vulnerability. These immune signatures, together with VCAM-1, may help characterize vascular vulnerability during the subclinical phase of plaque development.

**Supplementary Information:**

The online version contains supplementary material available at 10.1186/s12979-026-00588-2.

## Introduction

Cardiovascular disease (CVD) remains one of the leading causes of morbidity and mortality worldwide, with vascular aging representing a major determinant of risk [1, 2]. Age-related structural and functional changes in the arterial wall promote endothelial dysfunction and accelerate atherosclerosis development [3]. Importantly, this process begins long before clinical symptoms appear. During this silent phase, early vascular damage and subclinical atherosclerotic plaque (SAP) formation may already occur but remain undetected [4, 5]. Detecting such early alterations is therefore crucial for improving prevention and enabling timely intervention. Immunological mechanisms and inflammation are increasingly recognized as central drivers of atherogenesis. T cells play a pivotal role in maintaining chronic vascular inflammation. Both CD4^+^ and CD8^+^ T-cell subsets modulate plaque biology through pro- and anti-inflammatory effects [6–9], whereas regulatory T cells (Tregs) exert protective effects by limiting immune activation and stabilizing plaque [10]. Reduced Treg activity has been linked to accelerated vascular damage [8]. In addition, a broad network of mediators, including cytokines such as Interleukin (I)L-1β, IL-6, IL-18, and tumor necrosis factor-alpha (TNF-α), contributes to endothelial activation, arterial remodeling, and plaque progression [11–13]. Together with circulating proteins such as growth differentiation factor-15 (GDF-15) and osteoprotegerin (OPG), these immune-related pathways form a complex regulatory network that may provide early insight into emerging vascular vulnerability [14, 15]. Previous cross-sectional work in a carefully characterized cohort of healthy, medication-free older adults with high cardiorespiratory fitness demonstrated that SAP were accompanied by distinct cellular and molecular immune signatures. Individuals with SAP exhibited hallmarks of immunosenescence, including reduced proportions of naïve CD4^+^ T cells, expansion of differentiated subsets such as CD4^+^ and CD8^+^ CM cells, elevated inflammatory mediators, molecular features of cellular senescence, and alterations within Treg subpopulations [16, 17]. These findings underscored the pivotal role of immune system alterations in early plaque formation and suggested that immunological profiling may help identify individuals at increased vascular risk before clinical manifestation. Beyond immune dysregulation, lifestyle-related physiological factors also play a central role in vascular health. Both aerobic capacity [18, 19] and muscle strength [20] are well-established predictors of CVD risk and mortality. However, evidence linking these parameters to subclinical atherosclerosis remains limited and partially inconsistent. Recent data suggest that higher physical performance is associated with reduced SAP [21], indicating that functional capacity may modulate vascular aging. Physical activity is known to modulate immune aging by reducing chronic inflammation and immunosenescence by preserving naïve T cell pools, and limiting the accumulation of highly differentiated effector populations, thereby linking functional capacity directly to vascular immune health [22, 23]. Cross-sectional analyses cannot capture the temporal dynamics of disease development. It remains unclear which baseline characteristics identify individuals at heightened risk of future plaque onset and which longitudinal changes reflect biological processes associated with plaque development. Moreover, it is not known whether such associations persist independently of classical cardiovascular risk factors. These gaps highlight the need for longitudinal studies that integrate immune, metabolic and physiological domains to characterize subclinical vascular alterations before clinically manifest CVD emerges. To address this, we conducted a three-year study examining how adaptive immune remodeling, metabolic changes and functional capacity relate to the development of SAP. The study aimed to determine whether baseline immune and metabolic profiles are associated with subsequent plaque onset and whether within-person changes in these systems reveal biological changes associated with plaque initiation. This approach allowed us to investigate both potential determinants and dynamic processes associated with the early subclinical phase of plaque development in healthy older adults.

## Methods

### Study design and participants

**Fig. 1:**
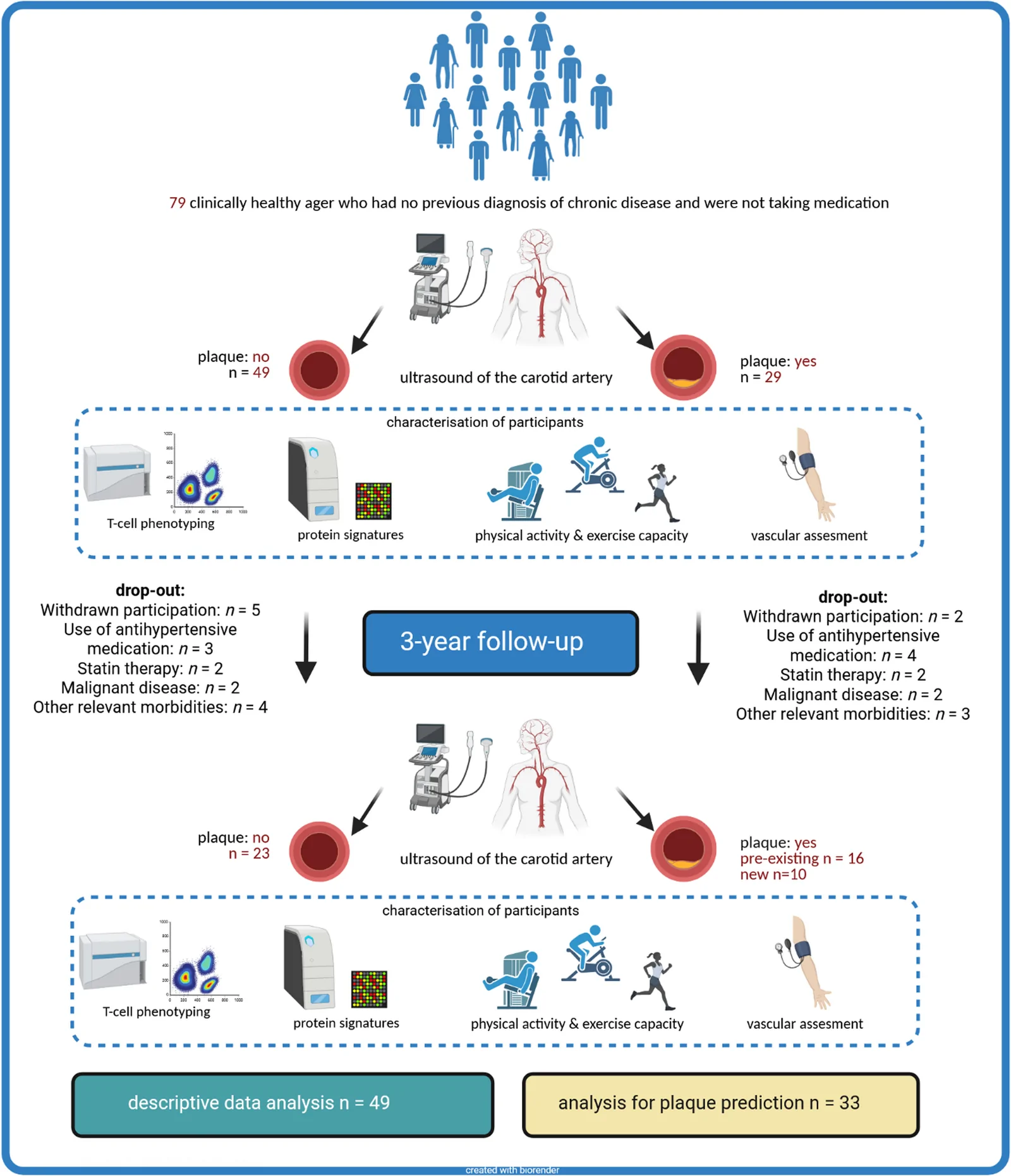
Overview of study design and participant flow. Seventy-nine participants underwent baseline assessments including vascular ultrasound, immune phenotyping, metabolic profiling and fitness testing. At 3-year follow-up, new subclinical atherosclerotic plaque (SAP) was assessed. Participants with complete longitudinal datasets (*n* = 49) were included in the descriptive longitudinal analyses. For plaque prediction analyses, individuals with pre-existing SAP were excluded, resulting in a final cohort of 33 participants (*n* = 23 no SAP; *n* = 10 with new SAP)

This longitudinal observational study was conducted between August 2020 and December 2024 as part of the Giessen Immune Aging Study. In total, 79 participants were enrolled at baseline. All participants were healthy, free of acute or chronic disease including infections and injuries and were taking no medication. A detailed description of the cohort and full inclusion and exclusion criteria has been provided previously by Böttrich et al. [17]. All participants were re-contacted three-years later and invited to undergo repeat screening assessments. Over the course of the follow-up period, several participants were excluded for health- or treatment-related reasons. In the plaque-free group, exclusions occurred due to withdrawn participation (*n* = 5), initiation of antihypertensive medication (*n* = 3), initiation of statin therapy (*n* = 2), malignant disease (*n* = 2), and other relevant morbidities (*n* = 4). In the group with plaque, exclusions occurred due to withdrawn participation (*n* = 2), initiation of antihypertensive medication (*n* = 4), initiation of statin therapy (*n* = 2), malignant disease (*n* = 2), and other newly emerging morbidities (*n* = 3). After applying all exclusion criteria, the final longitudinal cohort consisted of 49 participants (male: *n* = 30; female: *n* = 19) with a mean baseline age of 63.8 ± 3.8 years and a baseline BMI of 25.0 ± 3.1 kg/m². All remaining participants completed repeat carotid ultrasound screening at follow-up. At follow-up, participants were classified into three vascular groups based on carotid ultrasound findings: those who remained free of subclinical atherosclerotic plaque (no SAP), those who developed new plaque (new SAP), and those with plaque present at both time points (persistent SAP). This classification enabled two complementary analytical approaches. First, longitudinal trajectories were examined by quantifying within person changes in immune, metabolic and physiological variables across the three-year observation period. Second, to identify predictors of plaque initiation, baseline immune and metabolic profiles as well as three-year changes in these variables were evaluated in relation to incident SAP among participants who were plaque free at baseline. Incident plaque formation at follow-up served as the primary outcome for all prediction analyses. A detailed flow chart of participant allocation and follow-up is presented in Fig. 1, and baseline characteristics of the final study population are summarized in Table 1. Anthropometric measurements, immunological assessments, and fitness tests were conducted at the Department of Exercise Physiology and Sports Therapy, Justus-Liebig-University Giessen. Vascular screenings were performed at the University Hospital Giessen and Marburg, Giessen. The study was approved by the medical ethics committee of Justus-Liebig-University Giessen (AZ 100/20). All procedures conformed to the Declaration of Helsinki, and all participants provided written informed consent before enrolment.

**Table 1:** Baseline characteristics of participants categorized according to plaque status at baseline and follow-up

Characteristics	No SAP (*n* = 23)	New SAP (*n* = 10)	Persistent SAP (*n* = 16)	*p*-value
Age (years)	62.6 ± 3.6	64.6 ± 3.1	65.1 ± 4.0	0.088
Male n (%)	15 (65.2)	8 (80.0)	7 (43.8)	0.157
BMI (kg/m²)	24.2 ± 2.7	26.6 ± 4.0	25.1 ± 3.1	0.141
Body fat (%)	28.8 ± 6.3	27.6 ± 9.1	26.2 ± 6.6	0.548
Visceral fat (%)	31.3 ± 5.1	31.4 ± 8.4	29.4 ± 6.2	0.641
Systolic BP (mmHg)	123.5 ± 9.0	121.5 ± 9.2	130.8 ± 11.4	**0.035**
Diastolic BP (mmHg)	75.2 ± 10.0	71.4 ± 8.5	76.3 ± 8.6	0.413
VO_2_peak (ml/kg/min)	30.7 ± 6.6	28.9 ± 7.7	31.4 ± 7.7	0.678
Hand grip strength (kg)	42.4 ± 8.7	42.9 ± 9.6	46.9 ± 11.8	0.363
MET’s (min/week)	5271.2 ± 2885.2	3672.9 ± 1668.1	6061.7 ± 3328.8	0.126
Laboratory parameters				
Fasting glucose (mg/dl)	98.1 ± 7.9	95.7 ± 8.6	102.3 ± 11.1	0.185
Insulin (mU/l)	5.3 ± 2.0	6.8 ± 3.1	6.4 ± 3.2	0.274
HOMA-IR	1.3 ± 0.5	1.6 ± 0.8	1.6 ± 0.9	0.257
HbA1c (%)	5.6 ± 0.3	5.5 ± 0.38	5.6 ± 0.4	0.558
Total cholesterol (mg/dl)	227.2 ± 33.8	230.1 ± 35.9	204.1 ± 26.7	0.067
HDL-Cholesterol (mg/dl)	62.4 ± 16.1	57.1 ± 15.0	60.3 ± 12.2	0.639
LDL-Cholesterol (mg/dl)	150.3 ± 32.8	164.1 ± 34.2	142.6 ± 32.7	0.290
LDL-HDL-Ratio	2.6 ± 0.8	3.0 ± 0.9	2.5 ± 1.0	0.281
Cortisol (µg/dl)	16.1 ± 4.3	13.5 ± 2.8	15.2 ± 5.2	0.314
CMV + n (%)	12 (52.2)	5 (50.0)	9 (56.3)	0.952

All results presented as mean ± SD, % or n. p-value denotes differences in characteristics between participants with SAP, no SAP and persistent SAP

*Abbreviations*: *BMI *body mass index, *BP *blood pressure, *HDL *high-density lipoprotein, *HOMA-IR *Homeostatic Model Assessment for Insulin Resistance, *LDL *low-density lipoprotein, *SAP* subclinical atherosclerotic plaque

Bold *p*-values indicate statistically significant omnibus group effects (*p* < 0.05)

### Baseline characteristics

Venous fasting blood samples were collected from each participant between 08:00 and 10:00 for further analysis. These samples were used to determine standard laboratory parameters, peripheral blood mononuclear cells (PBMCs) and serum for protein signature analysis. Fasting concentrations of glucose and insulin, as well as total cholesterol, LDL, high-density lipoprotein (HDL), triglycerides, and cortisol level were determined in the serum using standard clinical laboratory methods by SYNLAB Medical Care Center (Bad Nauheim, Germany). Body fat percentage (%) and visceral adipose tissue (%) were assessed using bioelectric impedance analysis (BIA) with the BIACORPUS RX 4004 M and BodyComposition – Professional Version: 9.0.20413 software by MEDI CAL HealthCare GmbH.

### Carotid sonography and assessment of peripheral blood pressure

Carotid sonography was performed using a Philips cx50 device (Philips, Eindhoven, Netherlands) equipped with a linear transducer operating at a frequency range of 3–12 megahertz (MHz). Both sides of the carotid artery were examined in a cross-sectional B-mode, starting from the exit of the subclavian artery up to the bifurcation into the internal and external carotid arteries. The purpose was to identify subclinical atherosclerotic plaque (SAP) as yes or no. Multiple images of both the left and right common carotid arteries were obtained. A semi-automated edge-detection software was utilized to measure the intima-media thickness (IMT) over the distal wall of a common carotid artery segment that lies within 1–1.5 cm of the carotid bifurcation in the longitudinal view. The detection box analyzed a 10 mm length and was placed over the far wall of the common carotid artery segment located within 1–1.5 cm of the carotid bulb. Intima-media edges were determined, and IMT during end-diastole was calculated and expressed in millimeters. Prior to statistical analysis, carotid plaque was defined a priori using a prespecified threshold of ≥ 1.5 mm of IMT, in accordance with the recommendations of the American Society of Echocardiography [24]. Three frames on each side were analyzed offline by a single-blinded reader, and the mean values of all six IMT measurements for each patient were recorded and used for analysis. To acquire pulse pressure waveforms through oscillometry, we employed the non-invasive vascassist2^®^ device (isymed GmbH, Butzbach, Germany). The acquired pulse pressure waveforms were then analyzed using a validated electronic model of the arterial tree to assess vascular functional parameters. Parameters such as brachial systolic (SBP) and diastolic (DBP) blood pressure, were calculated.

### Isolation of peripheral blood mononuclear cells

To isolate peripheral blood mononuclear cells (PBMCs), fresh peripheral blood was diluted 1:1 with PBS (phosphate-buffered saline) and layered onto Lymphoprep density gradient medium using SepMate 50 mL tubes. After centrifugation at 1200×g for 10 min, the upper layer was carefully poured off. The isolated cells were then washed and centrifuged for 8 min at 300×g. Subsequently, the PBMCs were frozen down by resuspending them in freezing medium called Bambanker^®^. The frozen cells were stored at -80 °C for future analysis.

### T cell phenotyping by flow cytometry

To analyze T cells and their subsets, frozen PBMCs were thawed at 37 °C, washed twice in RPMI 1640 (Gibco, UK) containing 10% FBS (Gibco, UK), and then pelleted. The number of cells was divided in two samples according to two different panels used. For Panel 1 (Immune aging panel) and Panel 2 (Treg panel) fluorescence staining was performed directly after resting time. For panel 1 cells were resuspended in PBS at a concentration of 1 × 10⁶ cells/mL and stained with fluorescence Zombie Aqua fixable viability kit, CD4 (FITC), CD8 (AF700), CD197/CCR7 (BV421), CD45RA (APC) and CD57 (PE). For panel 2 cells were also resuspended in PBS at a concentration of 1 × 10⁶ cells/mL and stained with fluorescence Zombie Aqua fixable viability kit, CD3 (FITC), CD4 (AF700), CD25 (APC), CD45RO (PerCP/Cyanine5.5), CD197/CCR7 (BV421) and CD127 (PE) all from BioLegend (BioLegend Inc., San Diego, CA). For both panels the gating strategy was first selected lymphocytes based on forward scatter/side scatter (FSC/SSC) in a dot plot, with a minimum of 500,000 events per tube. Then singlets were identified within the lymphocyte gate, followed by the identification of live cells. For the Panel 1 CD4^+^ and CD8^+^ T cells were gated after determination of live cells (Zombie Aqua fixable viability kit) for CCR7-CD45RA to analyze the abundance of naïve T cells (CD45RA^+^CCR7^+^), effector memory (EM) T cells (CD45RA^−^CCR7^−^), central memory (CM) T-cells (CD45RA^−^CCR7^+^) and effector memory cells re-expressing CD45RA T cells (TEMRA) (CD45RA^+^CCR7^−^). Additionally, double-negative CD4^−^CD8^−^ lymphocytes were identified within the lymphocyte gate lacking both CD4 and CD8 expression after exclusion of non-viable cells. Terminal differentiated T cells were gated using CD57^+^. For the gating strategy to identify regulatory T cells the frequency of CD3^+^CD4^+^ cells were evaluated after determining live cells (Zombie Aqua fixable viability kit). T effector cells (Teff) were gated as CD4^+^CD25^+^CD127_high_, while Tregs were defined as CD4^+^CD25^+^CD127_low_. Within this population, Treg subsets were further gated as resting/naïve Tregs (rTregs; CD45RO^−^CCR7^+^) and effector-memory-like Tregs (mTregs; CD45RO^+^CCR7^−^), based on established surface marker-based classification of human Treg differentiation states [25]. In conclusion, frequencies of naïve, central memory, effector memory, EMRA, and CD57⁺ cells within the CD4⁺ and CD8⁺ compartments were expressed as percentages of their respective total CD4⁺ or CD8⁺ T-cell parent populations. Treg and Teff frequencies were expressed as percentages of total CD3⁺CD4⁺ T cells, whereas rTreg and mTreg frequencies were expressed as percentages of total Tregs. For detailed gating strategy, see Supplement Fig. S1 and S2. The ratio of Tregs to Teff was calculated by dividing the frequency of Tregs by the frequency of Teff cells and is reported as a dimensionless ratio. Flow cytometry analysis was performed using a CytoFLEX S (Beckman Coulter) and Kaluza analysis software 2.3 (Beckman Coulter), with compensation applied for spectral overlap when using multiple colors.

### Serum protein signatures and Cytomegalovirus (CMV) serostatus

Serum samples were stored at -80 °C until analysis. Proteins were determined using a human Magnetic Luminex Assay (Bio-Techne, Abingdon, Oxon, UK) and a Magpix Luminex instrument (Luminex Corp, Austin, TX, USA) according to manufacturer’s instructions. In total, 15 different proteins of inflammation or atherogenesis were determined for protein signature analysis: CCL-2, CXCL-9, -10, FGF-23, GDF-15, IL-1ra, -6, -10, 18, Osteopontin, Osteoprotegerin, TNF-α, TRAIL, VCAM-1, VEGF. Serum anti-CMV immunoglobulin G (IgG) antibodies were detected using a semiquantitative sandwich enzyme-linked immunosorbent assay (ELISA-Viditest anti-CMV IgG, VIDIA, Czech Republic). The procedure followed the manufacturer’s instructions. End-point optical density was measured by ELISA reader SPECTROstar ^®^ Nano (BMG Labtech, Germany).

### Measurements of CRF, muscle strength, physical activity and nutritional intakes

Cardiopulmonary exercise testing (CPET) was performed on an electronically braked cycle ergometer (Excalibur Sport^®^, Lode) using two standardized ramp protocols. Following a 3-min warm-up period without resistance, the specific protocol was selected according to the participant’s training and fitness status, aiming to reach maximal load within ~ 15 min. Prior to testing, participants were assessed for their sports history. Participants without systematic endurance training or competition experience completed a two-step ramp protocol: an initial 3-min unloaded phase, followed by cycling at 50 watts (W) with increments of 25 W every 3 min until 100 W, after which the workload was increased by 25 W every 2 min. Trained subjects started at 50 W after the 3-min warm-up period without load and increased by 50 W every 3 min. The test was performed until complete exhaustion. The following criteria were used to verify exhaustion: request of the participant due to extreme tiredness and/or perception of the intense dyspnea; reached the maximum heart rate (HR) predicted by age (HR max) was ≥ 85%; peak respiratory exchange ratio RER > 1.1; VO_2_ plateau was reached even with increasing workload. Ventilatory and metabolic parameters were collected by respiration using Metalyzer 3-B (Cortex, Germany) and were analyzed. The average of the last test 30 s was used to determine the VO_2_peak. Maximum voluntary grip strength of the dominant hand was measured using a hand grip dynamometer (Baseline^®^ Hydraulic Hand Dynamometer LiTE^®^, Fabrication Enterprises Inc., US). Participants were instructed to keep their arms by their side during the assessment. Verbal encouragement was provided to motivate the participants to exert their maximum effort. Three maximal contractions were performed, and the highest recorded grip strength value was used for subsequent analysis. Physical activity was determined by using a self-administered form of the international physical activity questionnaire (IPAQ) long form. The questionnaire assesses physical activity (PA) performed during the preceding seven days. Participants were asked to report all activities performed for at least 10 min during that period. Participants subsequently reported the number of days per week and duration per day for each activity. The energy expenditure indicator metabolic equivalent of task (MET) per week was calculated by multiplying the number of minutes per week of each activity-based metabolic cost.

### Data analysis

All statistical analyses were performed using IBM SPSS Statistics (Version 29.0, IBM Corp., Armonk, NY, USA) and GraphPad Prism (Version 10, GraphPad Software, San Diego, CA, USA) for graphical visualization. Descriptive statistics were generated for the total cohort (*n* = 49) and for the three vascular groups (No SAP, New SAP, Persistent SAP). Continuous variables are reported as mean ± standard deviation (SD) and categorical variables as absolute and relative frequencies. To assess temporal changes and potential group differences across the 3-year observation period, two-factor mixed-design repeated-measures ANOVA was applied, allowing evaluation of main effects of time, and group × time interactions for all immunological, metabolic, and physiological variables. Where significant effects were detected, post hoc comparisons were conducted using Bonferroni–Holm correction to identify relevant time points and intergroup differences. Analyses predicting incident plaque formation focused on participants who were plaque-free at baseline, comparing those who remained free of plaque with those who developed new SAP at follow-up. To identify early determinants of vascular risk, univariate binary logistic regression analyses were performed for all immunological, metabolic, physiological, and clinical variables at baseline, using incident plaque formation (yes/no) as the outcome. To evaluate predictive value of temporal changes, additional univariate models were run using delta values calculated as follow-up minus baseline (Δ = follow-up – baseline). For both the baseline and Δ models, the ten variables with the lowest p-values were retained for visualization in forest plots, displaying odds ratios (OR) and 95% confidence intervals (CI). To determine independent predictors of incident plaque formation, stepwise multivariable logistic regression analyses were conducted separately for baseline predictors and longitudinal changes. Primary variables entered into each model were the immunological and serological parameters that ranked among the top ten predictors in the respective univariate analyses. To minimize multicollinearity, only one covariate per physiological domain was included, selected based on the strongest univariate association within each category: body composition, glucose metabolism, age, blood lipids, physical performance/activity, and blood pressure. Covariates were entered stepwise in order of statistical relevance (ascending p-value). Serum protein concentrations and physical activity values were log-transformed and standardized before logistic regression analysis. A two-sided p-value < 0.05 was considered statistically significant. Given the exploratory nature of the study, no additional adjustment for multiple testing was applied.

## Results

Over the three-year observation period, new SAP developed in 30.3% of participants who were plaque-free at baseline, while the overall plaque prevalence reached 53% at follow-up. Anthropometric measures, classical clinical laboratory parameters, and blood pressure values were compared retrospectively across the three groups (no SAP, new SAP, and persistent SAP) (Table 1). Baseline clinical and anthropometric characteristics were broadly comparable across the three groups. An overall group effect was observed for systolic blood pressure. However, none of the pairwise post hoc comparisons reached statistical significance.

### Changes in anthropometric and clinical parameters over time

**Fig. 2:**
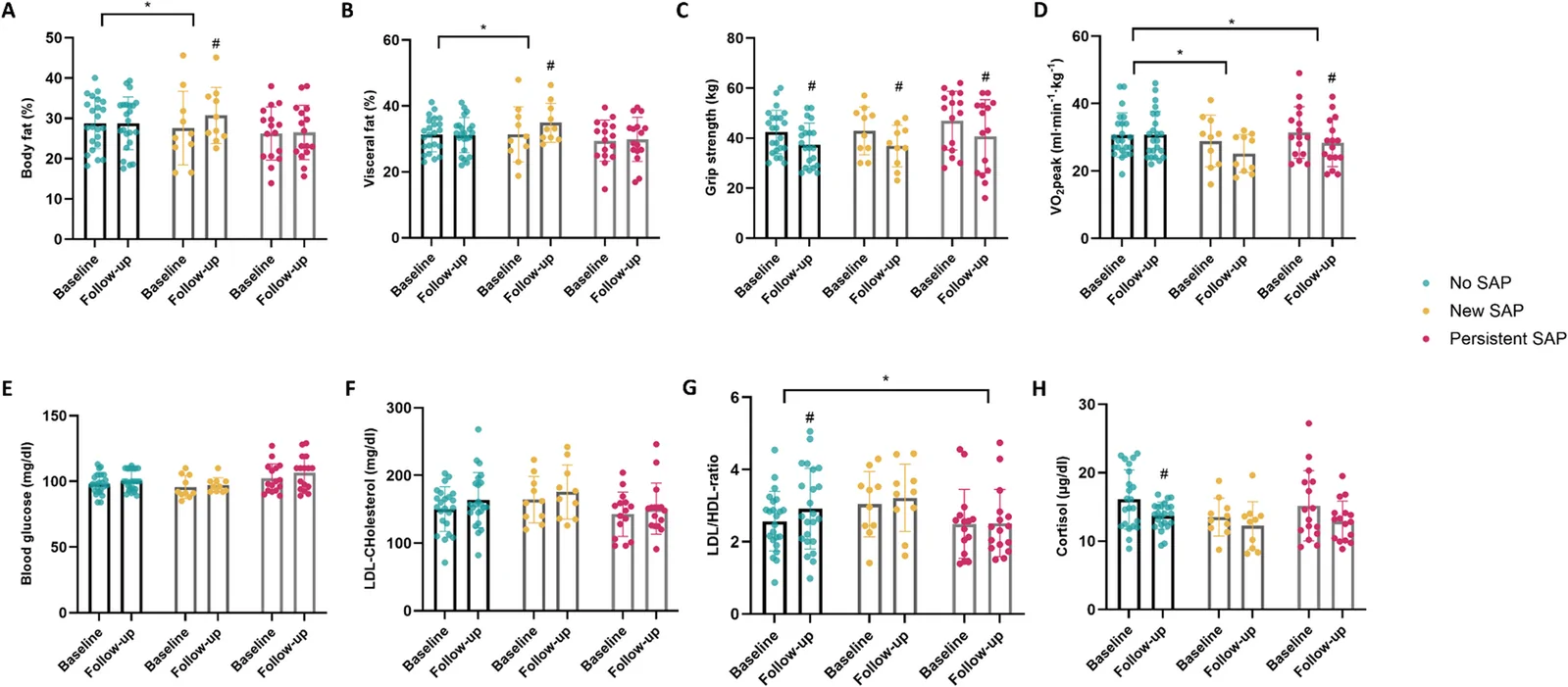
Longitudinal changes in anthropometric, metabolic, and performance parameters across subclinical atherosclerotic plaque (SAP) groups. Participants were categorized into three vascular groups based on carotid ultrasound at baseline and follow-up: No SAP (subclinical atherosclerotic plaque absent at both time points; teal), New SAP (incident plaque formation at follow-up; yellow), and Persistent SAP (plaque present at baseline and follow-up; magenta). Bar graphs display group means ± SD with individual data points overlaid. Panels depict changes from baseline to follow-up in body fat percentage (**A**), visceral fat percentage (**B**), hand grip strength (**C**), VO_2_peak (**D**), fasting blood glucose (**E**), LDL cholesterol (**F**), LDL/HDL ratio (**G**), and cortisol (**H**). Significant time effects within groups are indicated by # (*p* < 0.05). * denote interaction effects or significant differences in time × group comparisons across the observation period (*p* < 0.05)

Two-way mixed-design repeated measures ANOVA showed a significant main effect of time (F(1,46) = 6.30, *p* = 0.016) and a time × group interaction (F(2,46) = 4.30, *p* = 0.019) for body fat (%). Post hoc analyses showed an increase in the new SAP group (*p* = 0.026). The interaction was driven by a steeper increase in new SAP group compared with no SAP group (*p* = 0.021). For visceral fat (%), results indicated a main effect of time (F(1,46) = 6.01, *p* = 0.018) as well as a time × group interaction (F(2,46) = 4.28, *p* = 0.020). Post hoc analyses showed an increase in the new SAP group (*p* = 0.027). The post hoc analysis reflected an increase in the new SAP group compared with the no SAP group (*p* = 0.018). In VO_2_peak, a main effect of time (F(1,46) = 14.79, *p* < 0.001) and a time × group interaction (F(2,46) = 4.89, *p* = 0.012) were observed. Post hoc analyses showed a decline in the persistent SAP group (*p* = 0.034) and a trend in new SAP (*p* = 0.070). The interaction showed differences in the development of VO_2_peak between the no SAP group and both the new SAP (*p* = 0.038) and the persistent SAP groups (*p* = 0.038). For hand grip strength, the analysis yielded a main effect of time (F(1,43) = 89.32, *p* < 0.001). Post hoc analyses indicated a decline in all groups (no SAP: *p* < 0.001; new SAP: *p* = 0.001; persistent SAP: *p* < 0.001). Blood glucose levels also showed a main effect of time (F(1,46) = 4.49, *p* = 0.040). For LDL cholesterol (mg/dl), a main effect of time was detected (F(1,45) = 5.23, *p* = 0.027). For both, post hoc analyses did not show pairwise differences. The LDL/HDL ratio exhibited a significant main effect of time (F(1,45) = 5.94, *p* = 0.019) as well as a significant time × group interaction (F(2,45) = 3.77, *p* = 0.031). Post hoc analyses indicated an increase only in the ratio within the no SAP group (*p* = 0.012). The interaction reflected a differential trajectory over time, with differences between no SAP and persistent SAP groups (*p* = 0.026). Cortisol levels (µg/dl) showed a main effect of time (F(1,45) = 11.14, *p* = 0.002). Post hoc analyses indicated a decrease within the no SAP group (*p* = 0.018). In addition, all other anthropometric parameters, blood pressure and conventional clinical blood markers did not show significant changes over time or between groups (Fig. 2). Full descriptive statistics are provided in Supplementary Tab. S1-S2.

### Changes in CD4^+^ T cell subsets

**Fig. 3:**
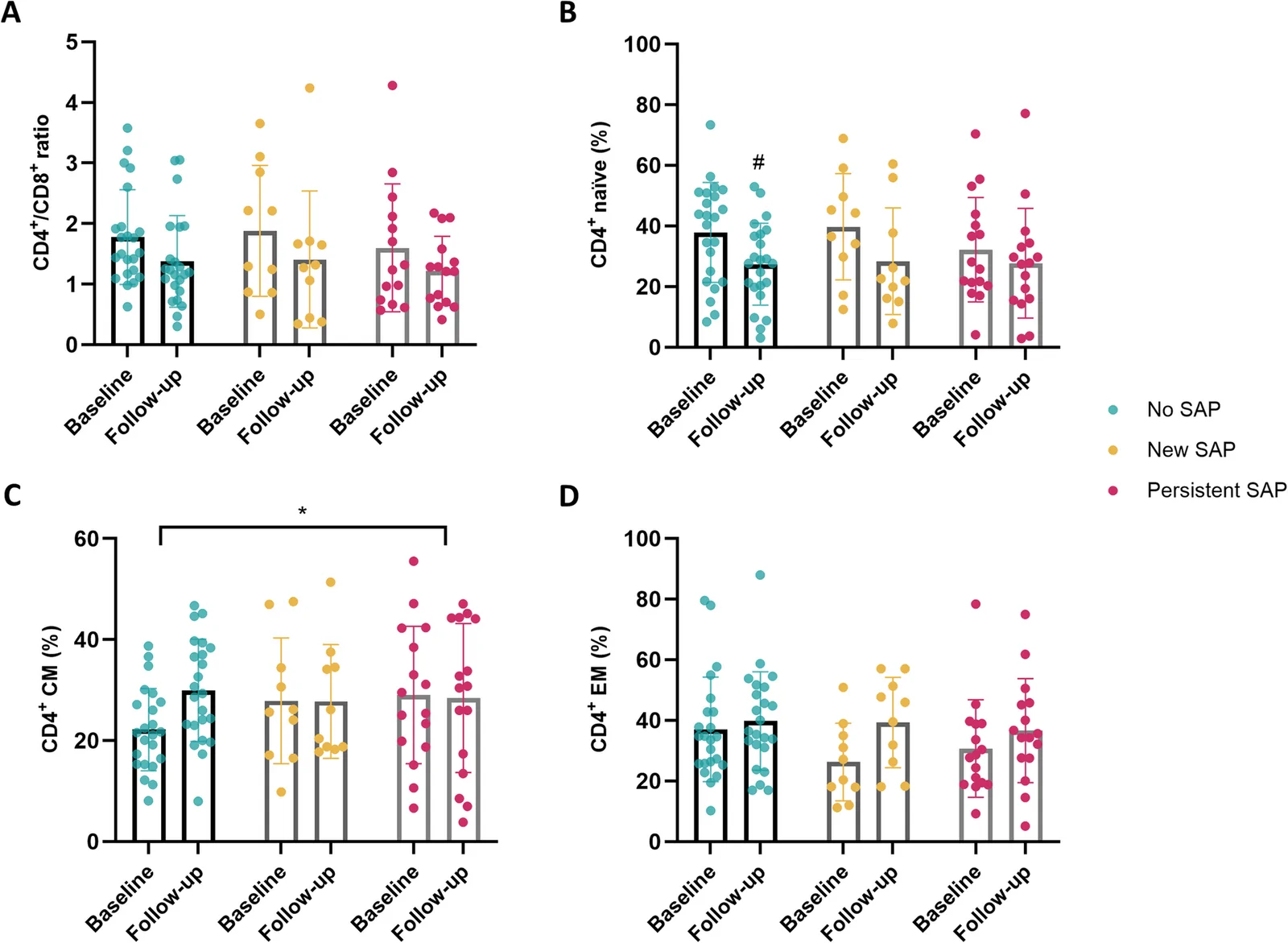
Longitudinal changes in CD4^+^/CD8^+^ ratio and CD4^+^ T cell subpopulation frequencies across subclinical atherosclerotic plaque (SAP) groups. CD4^+^ T-cell subsets are expressed as percentages of their respective total CD4^+^ T-cell parent populations. Participants were categorized into three vascular groups based on carotid ultrasound at baseline and follow-up: No SAP (subclinical atherosclerotic plaque absent at both time points; teal), New SAP (incident plaque formation at follow-up; yellow), and Persistent SAP (plaque present at baseline and follow-up; magenta). Bar graphs display group means ± SD with individual data points overlaid. Panels depict changes from baseline to follow-up in CD4^+^ /CD8^+^ ratio (**A**), CD4^+^ naïve T cells (**B**), CD4^+^ central memory (CM) T cells (**C**) and CD4^+^ effector memory (EM) T cells (**D**), Significant time effects within groups are indicated by # (*p* < 0.05). * denote interaction effects or significant differences in time × group comparisons across the observation period (*p* < 0.05)

For the CD4^+^/CD8^+^ ratio, the analysis indicated a main effect of time (F(1,46) = 8.46, *p* = 0.006). The ratio declined over the observation period, but post hoc comparisons did not identify differences. CD4^+^ naïve T cells also showed a time effect (F(1,46) = 11.86, *p* = 0.001). Post hoc testing indicated a within-group reduction in the no SAP group (*p* = 0.015). For CD4^+^ CM T cells the time × group interaction reached significance (F(2,46) = 4.28, *p* = 0.020). Post hoc analyses indicated a difference between the no SAP and the persistent SAP groups (*p* = 0.035). For CD4^+^ EM T cells, a time effect was observed (F(1,46) = 5.64, *p* = 0.022). Post hoc analyses did not reveal group-specific changes. Data are shown in Fig. 3 and full descriptive statistics are provided in Supplementary Tab. S3.

### Changes in CD8^+^ T cell subsets

**Fig. 4:**
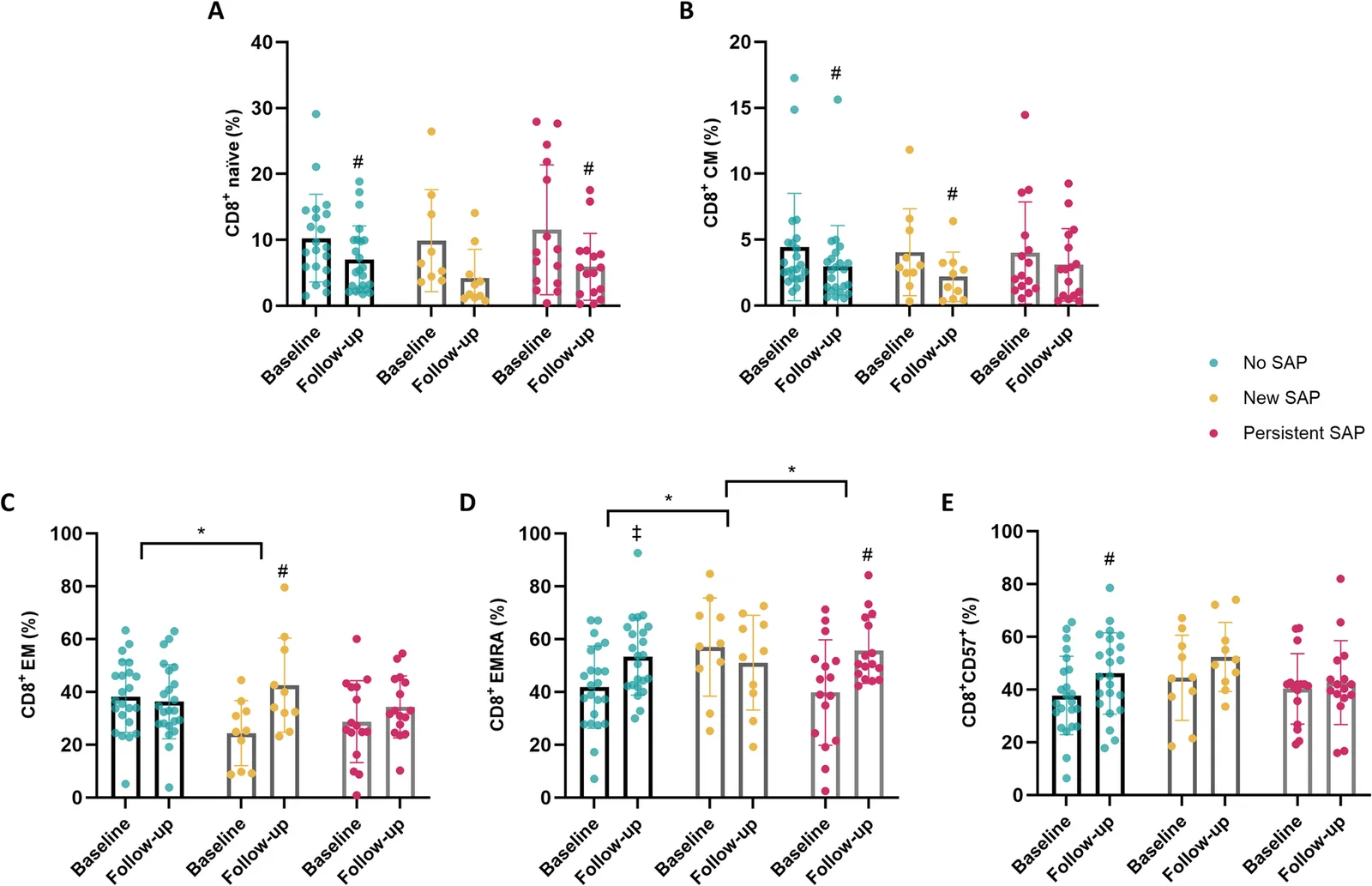
Longitudinal changes in CD8^+^ T cell subpopulation frequencies across subclinical atherosclerotic plaque (SAP) groups. CD8^+^ T-cell subsets are expressed as percentages of their respective CD8^+^ T-cell parent populations. Participants were categorized into three vascular groups based on carotid ultrasound at baseline and follow-up: No SAP (subclinical atherosclerotic plaque absent at both time points; teal), New SAP (incident plaque formation at follow-up; yellow), and Persistent SAP (plaque present at baseline and follow-up; magenta). Bar graphs display group means ± SD with individual data points overlaid. Panels depict changes from baseline to follow-up in CD8^+^ naïve T cells (**A**), CD8^+^ central memory (CM) T cells (**B**), CD8^+^ effector memory (EM) T cells (**C**), and CD8^+^ effector memory re-expressing CD45RA T cells (**D**) and CD8^+^CD57^+^ T cells (**E**). Significant time effects within groups are indicated by # (*p* < 0.05) and ǂ (*p* < 0.01). * denote interaction effects or significant differences in time × group comparisons across the observation period (*p* < 0.05)

For CD8^+^ naïve T cells, the analysis indicated a main effect of time (*F*(1,46) = 17.24, *p* < 0.001). Post hoc analyses confirmed within-group declines in the no SAP (*p* = 0.036) and persistent SAP groups (*p* = 0.036). A similar pattern was observed for CD8^+^ CM T cells, where a time effect emerged (*F*(1,46) = 8.22, *p* = 0.006). Frequencies declined in the no SAP (*p* = 0.036) and new SAP (*p* = 0.034). In CD8^+^ EM T cells, both a main effect of time (*F*(1,44) = 7.78, *p* = 0.008) and a time × group interaction (*F*(2,44) = 4.67, *p* = 0.014) were observed. While values remained stable in the no SAP and persistent SAP group, a marked rise was observed in the new SAP group (*p* = 0.018). Post hoc tests confirmed differences between no and new SAP (*p* = 0.011). For CD8^+^ EMRA T cells, the analysis showed both a main effect of time (*F*(1,46) = 6.41, *p* = 0.015) and an interaction (*F*(2,46) = 4.48, *p* = 0.017). Increases were observed in the no SAP group (*p* = 0.006) and the persistent SAP group (*p* = 0.014). Post hoc analyses confirmed differential developments between these groups (*p* = 0.035) as well as between the new and persistent SAP groups (*p* = 0.017). Finally, CD8^+^CD57^+^ T cells displayed a significant overall time effect (*F*(1,46) = 10.53, *p* = 0.002). Levels increased over time, with a significant within-group rise detected in the no SAP group (*p* = 0.007). Data are shown in Fig. 4, representative flow cytometry plots are provided in Fig. S3 and full descriptive statistics are provided in Supplementary Tab. S3.

### Dynamics of regulatory T cell compartments

**Fig. 5:**
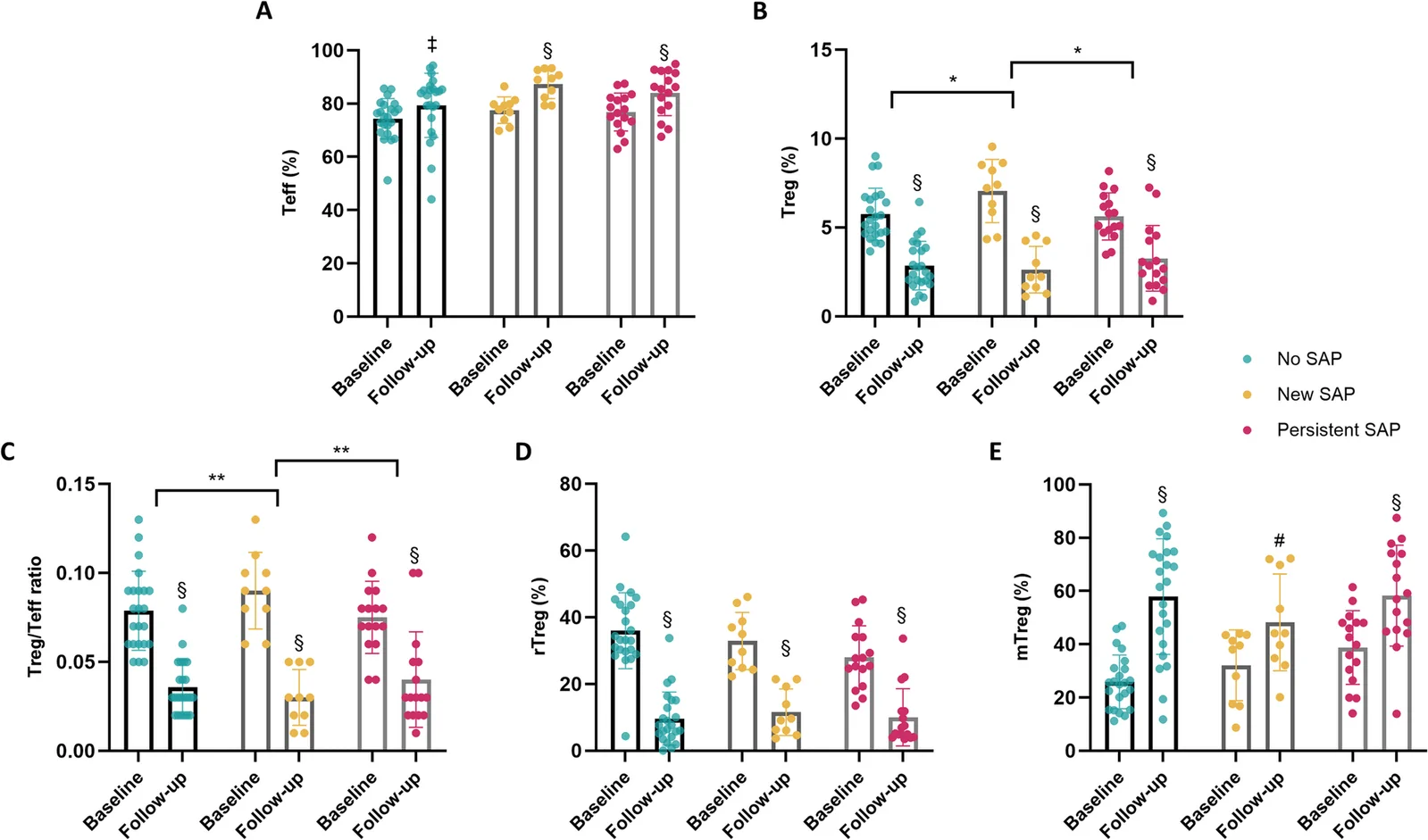
Longitudinal changes in T effector cell frequencies (Teff), frequencies of regulatory T cells (Treg) as well as their subpopulation and Treg/Teff ratio across subclinical atherosclerotic plaque (SAP) groups. Treg and Teff frequencies are expressed as percentages of total CD3^+^CD4^+^ T cells, whereas rTreg and mTreg frequencies are expressed as percentages of total Tregs. Participants were categorized into three vascular groups based on carotid ultrasound at baseline and follow-up: No SAP (subclinical atherosclerotic plaque absent at both time points; teal), New SAP (incident plaque formation at follow-up; yellow), and Persistent SAP (plaque present at baseline and follow-up; magenta). Bar graphs display group means ± SD with individual data points overlaid. Panels depict changes from baseline to follow-up in Teff frequencies (**A**), Treg frequencies (**B**), Treg/Teff ratio (**C**), resting/naïve (r)Tregs (**D**) and effector-memory-like (m)Tregs (**E**). Significant time effects within groups are indicated by # (*p* < 0.05), ǂ (*p* < 0.01) and § (*p* < 0.001). * denote interaction effects or significant differences in time × group comparisons across the observation period (*p* < 0.05) and ** (*p* < 0.01)

In Teff population, a time effect was detected (*F*(1,46) = 32.59, *p* < 0.001). Frequencies increased in all groups (no SAP: *p* = 0.005; new SAP and persistent SAP: both *p* < 0.001). For Tregs, the analysis yielded a main effect of time (*F*(1,46) = 137.39, *p* < 0.001) together with a time × group interaction (*F*(2,46) = 4.11, *p* = 0.023). Frequencies declined in all groups (*p* < 0.001). Between-group comparisons further indicated differences in the trajectories of change between the no SAP and new SAP groups (*p* = 0.005) as well as between the new and persistent SAP groups (*p* = 0.021). The Treg/Teff ratio also displayed a time effect (*F*(1,43) = 202.63, *p* < 0.001) and an interaction (*F*(2,43) = 6.75, *p* = 0.003). Ratios decreased across all groups (*p* < 0.001). Additional comparisons showed differences between the no and new SAP groups (*p* = 0.008) as well as between the new and persistent SAP groups (*p* = 0.002). For rTregs, a time effect was observed (*F*(1,46) = 179.59, *p* < 0.001). Frequencies decreased in all groups (*p* < 0.001). Finally, mTregs showed a main effect of time (*F*(1,46) = 42.15, *p* < 0.001). Frequencies increased consistently across groups (no SAP: *p* < 0.001; new SAP: *p* = 0.049; persistent SAP: *p* < 0.001). Data are shown in Fig. 5, representative flow cytometry plots are provided in Fig. S3 and full descriptive statistics are provided in Supplementary Tab. S3. To examine whether these longitudinal changes were associated with age, exploratory Spearman correlation analyses were performed using baseline age. No significant associations were observed.

### Changes in circulating serum proteins and cytokines

**Fig. 6:**
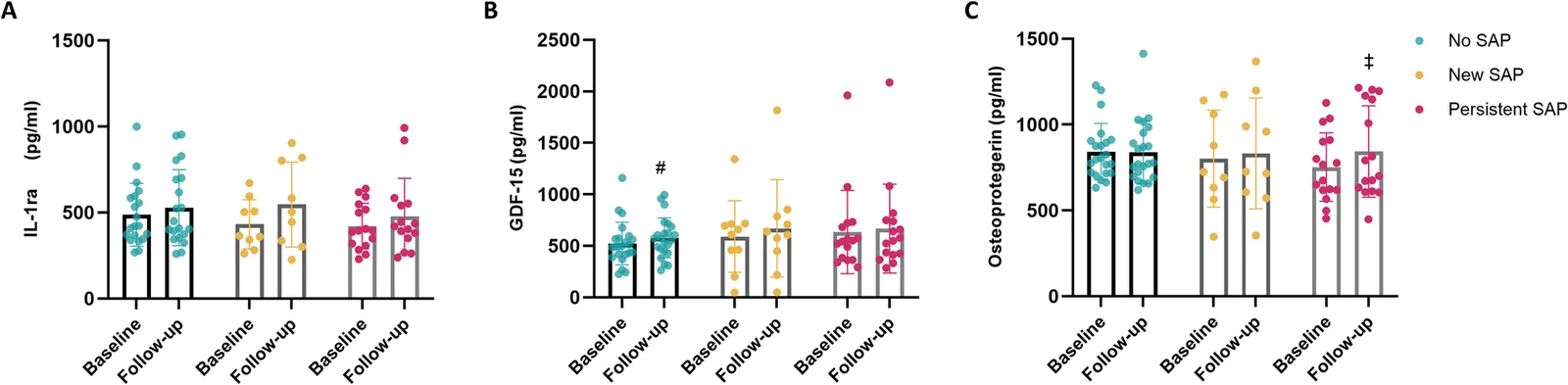
Longitudinal changes in serological markers across subclinical atherosclerotic plaque (SAP) groups. Participants were categorized into three vascular groups based on carotid ultrasound at baseline and follow-up: No SAP (subclinical atherosclerotic plaque absent at both time points; teal), New SAP (incident plaque formation at follow-up; yellow), and Persistent SAP (plaque present at baseline and follow-up; magenta). Bar graphs display group means ± SD with individual data points overlaid. Panels depict changes from baseline to follow-up in Interleukin- (IL-)1 receptor antagonist (ra) (**A**), growth differentiation factor-(GDF-) 15 (**B**) and Osteoprotegerin (**C**). Significant time effects within groups are indicated by # (*p* < 0.05) and ǂ (*p* < 0.01)

For GDF-15, two-way repeated-measures ANOVA revealed a main effect of time (*F*(1,46) = 11.76, *p* = 0.001). Serum concentrations increased in no SAP (*p* = 0.012). IL-1ra also showed a time effect (*F*(1,46) = 5.63, *p* = 0.022), although post hoc testing did not reveal significant pairwise differences. For osteoprotegerin, a main effect of time (*F*(1,46) = 6.54, *p* = 0.014) was observed. Serum levels increased significantly in the persistent SAP group (*p* = 0.003). All other assessed serum proteins did not show significant changes over time or between groups. Data are shown in Fig. 6 and full descriptive statistics are provided in Supplementary Tab. S4.

### Baseline determinants and longitudinal changes as predictors of new plaque development

**Fig. 7:**
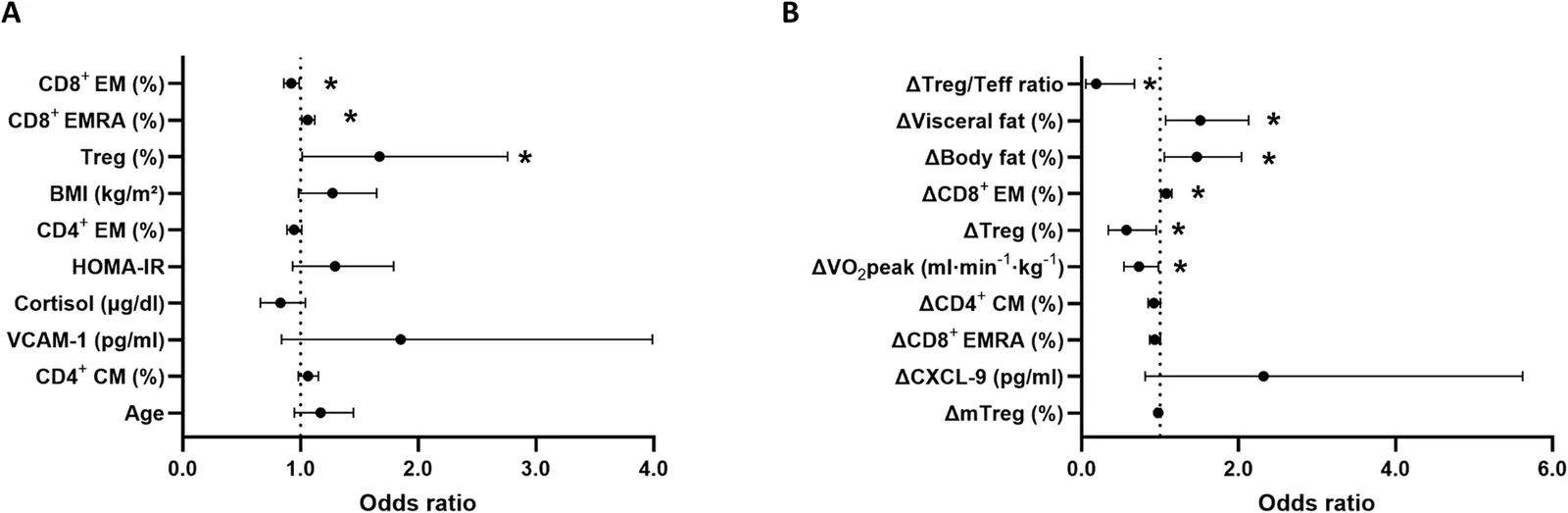
Top predictors of new subclinical atherosclerotic plaque (SAP) development at baseline and across longitudinal changes. Displayed are the top 10 predictors ranked by ascending p-values, shown as odds ratios (OR) with 95% confidence intervals. Statistically significant associations are marked with an asterisk * (*p* < 0.05). **A** Baseline immune, metabolic and clinical parameters associated with the development of new subclinical atherosclerotic plaque (SAP) formation over the 3-year observation period. **B** Longitudinal changes (Δ) in immune, metabolic and clinical parameters associated with the risk of new SAP formation over the 3-year observation period. Abbreviations: Δ (delta): indicates change over the 3-year period (follow-up minus baseline), BMI: body mass index, CM: central memory, CXCL-9: C-X-C motif chemokine ligand 9, EM: effector memory, EMRA: effector memory re-expressing RA, HOMA-IR: homeostatic model assessment of insulin resistance, mTreg: memory regulatory T cells, Treg: regulatory T cells, VCAM-1: vascular cell adhesion molecule-1, VO_2_peak: peak oxygen uptake

To explore early predictors of incident SAP, all immune, metabolic and physiological variables were first examined using univariate logistic regression. Predictors were ranked by p value, and the ten strongest baseline and longitudinal markers were selected for detailed presentation, regardless of statistical significance. Based on this ranking, the top baseline predictors included several immune parameters that differed in their association with incident SAP, as shown in Fig. 7A. Among T cell subsets, lower frequencies of CD8^+^ EM T cells (OR = 0.92, 95% CI [0.85–0.99], *p* = 0.022) and higher frequencies of CD8^+^ EMRA T cells (OR = 1.06, 95% CI [1.01–1.11], *p* = 0.033) were significantly associated with plaque development. Additionally, higher baseline levels of Tregs predicted SAP occurrence (OR = 1.67, 95% CI [1.01–2.76], *p* = 0.045). Trends toward elevated risk were also observed for BMI (OR = 1.27, 95% CI [0.98–1.65], *p* = 0.067), the HOMA-IR (OR = 2.49, 95% CI [0.70–8.82], *p* = 0.121), VCAM-1 (OR = 1.96, 95% CI [0.83–3.98], *p* = 0.126) and baseline age (OR = 1.17, 95% CI [0.95–1.45], *p* = 0.145), whereas higher CD4^+^ EM T cell frequencies (OR = 0.95, 95% CI [0.89–1.01], *p* = 0.101) or cortisol levels (OR = 0.83, 95% CI [0.66–1.04], *p* = 0.108) showed a tendency toward a protective association. Full univariate results are provided in Supplementary Tab. S5.

When examining changes (Δ) over the three-year follow-up period, several of the highest ranked delta predictors, reflecting alterations in immune status, body composition and fitness, were associated with the development of new SAP (Fig. 7B). Greater preservation of the Treg/Teff ratio was associated with lower odds of new SAP (OR = 0.19, 95% CI [0.05–0.67], *p* = 0.010). Conversely, a greater decline was associated with higher odds of incident SAP. Also, an increase in CD8^+^ EM T cells predicted new SAP (OR = 1.08, 95% CI [1.01–1.15], *p* = 0.027), and in addition a decrease in Treg was associated with higher odds of new SAP (OR = 0.57, 95% CI [0.34–0.95], *p* = 0.031). An increase in body fat (%) (OR = 1.47, 95% CI [1.06–2.04], *p* = 0.023) and visceral fat (%) (OR = 1.51, 95% CI [1.07–2.13], *p* = 0.018) were both linked to a higher likelihood of new SAP. Conversely, improvements in cardiorespiratory fitness (ΔVO_2_peak) were associated with a reduced risk of new SAP (OR = 0.73, 95% CI [0.54–0.98], *p* = 0.035). Additional trends were noted for CD4^+^ CM (OR = 0.92, 95% CI [0.85–1.00], *p* = 0.054), CD8^+^ EMRA T cells (OR = 0.93, 95% CI [0.87–1.00], *p* = 0.061) and mTreg (OR = 0.98, 95% CI [0.95–1.01], *p* = 0.118. A non-significant trend toward higher odds of new SAP was observed for increasing CXCL-9 levels (OR = 2.32, 95% CI [0.81–5.62], *p* = 0.117). Full univariate delta results are provided in Supplementary Tab. S6.

### Multivariable logistic regression models of baseline parameters and longitudinal changes associated with incident SAP

In stepwise multivariable logistic regression analyses, immunological and metabolic parameters were evaluated as predictors of new SAP formation. Across all baseline models, the inclusion of conventional cardio-metabolic covariates (BMI, HOMA-IR, age, LDL/HDL ratio, systolic blood pressure and METs) moderately improved overall model fit (χ² range = 6.0–23.4), although only a subset of markers retained independent significance after full adjustment (Table 2). CD8^+^ EM T cells remained inversely associated with new SAP across all models (Supplementary Tab. S7), and this association persisted after full adjustment in Model 6 (*p* = 0.042). CD8^+^ EMRA T cells showed a positive association with plaque formation in early models up to Model 5 (*p* = 0.040), but the effect attenuated and lost significance in the fully adjusted model (Supplementary Tab. S8). Tregs demonstrated a positive association with plaque presence in initial models (Model 1–2; *p* = 0.044), but this signal did not persist once additional metabolic and cardiovascular covariates were included (Supplementary Tab. S9). In contrast, VCAM-1 consistently predicted plaque development across all baseline models, remaining a strong independent predictor even after full adjustment (Model 6; *p* = 0.025) (Supplementary Tab. S11). No significant associations were observed for CD4^+^ EM (Supplementary Tab. S10) or CD4^+^ CM (Supplementary Tab. S12) T cell subsets after adjustment.

**Table 2:** Multivariable logistic regression models assessing immune and metabolic predictors of new subclinical atherosclerotic plaque

	CD8 + EM (Model 6)	CD8 + EMRA (Model 5)	Treg (Model 2)	VCAM-1 (Model 6)
	OR (95% CI)	OR (95% CI)	OR (95% CI)	OR (95% CI)
Primary variable	0.92 (0.84–0.99)*	1.14 (1.01–1.30)*	1.88 (1.02–3.46)*	173.02 (1.91–15644.46)*
BMI	1.33 (0.78–2.25)	1.61 (0.90–2.88)	1.30 (0.94–1.81)	2.30 (0.97–5.42)
HOMA-IR	1.54 (0.17–14.1)	0.52 (0.04–6.81)	1.28 (0.21–7.88)	0.11 (0.02–6.80)
Age	1.21 (0.93–1.58)	1.14 (0.88–1.47)	-	1.12 (0.81–1.55)
LDL/HDL-ratio	0.97 (0.18–5.27)	1.23 (0.26–5.89)	-	8.34 (0.51–136.46)
MET’s	0.67 (0.11–3.98)	0.59 (0.09–3.82)	–	0.01 (0.00–1.31)
Systolic BP	0.92 (0.79–1.08)	-	–	1.08 (0.88–1.33)
Χ²	13.90*	15.79*	8.74*	23.41***

Odds ratios (OR) with 95% confidence intervals (CI) for the primary immune variables (CD8⁺ EM, CD8⁺ EMRA, Treg, VCAM-1) and covariates across four regression models. Model fit is indicated by χ² statistics

*Abbreviations*: *BMI *body mass index, *BP *blood pressure, *CI *confidence interval, *EM *effector memory, *EMRA *effector memory re-expressing RA, *HDL *high-density lipoprotein, *HOMA-IR *homeostatic model assessment of insulin resistance, *LDL *low-density lipoprotein, *MET’s *metabolic equivalents of task, *OR *odds ratio, *Treg *regulatory T cells, *VO*_*2*_*peak* peak oxygen uptake, *VCAM-1 *vascular cell adhesion molecule-1, *Χ² *chi-square

Asterisks denote significance levels (*p < 0.05; ***p < 0.001)

In the models examining longitudinal change variables (Δ), several immunological and metabolic adaptations were assessed as predictors of new SAP. As with the baseline analyses, only a subset of variables retained independent significance after full adjustment (Table 3). Increases in CD8 + EM cells were positively associated with incident SAP across the Δ models and remained independently associated in the fully adjusted Model 6 (*p* = 0.048). Greater preservation of the Treg/Teff ratio was inversely associated with incident SAP in Models 1 and 2. Conversely, a greater decline was associated with higher odds of incident SAP but this association did not remain significant following covariate adjustment. No significant associations were observed for ΔTreg, ΔCD8^+^ EMRA, ΔCD4^+^ CM or ΔCXCL 9 after inclusion of covariates (Supplementary Tab. S15–S19). Increases in visceral fat (%) emerged as a covariate-dependent risk factor in models including ΔCXCL 9 (*p* = 0.027), though this effect diminished with full adjustment. Sex and CMV serostatus were additionally tested in all multivariable models but did not yield significant associations (data not shown).

**Table 3:** Multivariable logistic regression models assessing longitudinal changes in immune, metabolic and fitness parameters as predictors of new subclinical atherosclerotic plaque

	ΔCD8 + EM (Model 6)	ΔTreg/Teff ratio (Model 2)	ΔCXCL9 (Model 1)
	OR (95% CI)	OR (95% CI)	OR (95% CI)
Primary variable	1.20 (1.00–1.46)*	0.28 (0.08–0.99)*	2.15 (0.67–6.89)
ΔVisceral fat	0.93 (0.48–1.80)	1.21 (0.80–1.82)	1.47 (1.04–2.08)*
ΔVO_2_peak	0.32 (0.06–1.66)	0.91 (0.62–1.34)	-
Age baseline	1.51 (0.73–2.89)	-	-
ΔHOMA-IR	0.57 (0.03–12.02)	-	–
ΔLDL/HDL ratio	0.02 (0.00–1.49)	–	–
ΔSystolic BP	0.90 (0.76–1.06)	–	–
Χ²	26.25***	12.86**	11.29**

Odds ratios (OR) with 95% confidence intervals (CI) are shown for the primary change variables (ΔCD8⁺ EM, ΔTreg/Teff ratio, ΔCXCL9) and covariates across three regression models. Model fit is indicated by χ² statistics

*Abbreviations*: *BP *blood pressure, *CI *confidence interval, *CXCL9 *C-X-C motif chemokine ligand 9, *EM *effector memory, *HDL *high-density lipoprotein, *HOMA-IR *homeostatic model assessment of insulin resistance, *LDL *low-density lipoprotein, *OR *odds ratio, *Teff *effector T cells, *Treg *regulatory T cells, *VO*_*2*_*peak* peak oxygen uptake, *Χ² *chi-square

Asterisks denote significance levels (*p < 0.05; **p < 0.01; ***p < 0.001)

## Discussion

Over the three-year observation period, SAP increased by about 30% within our cohort, with new plaques appearing in one third of previously plaque-free participants and an overall prevalence of 53% at follow-up. Beyond this structural progression, distinct time-dependent changes were observed in body composition, cardiorespiratory fitness, and immune profiles. At the immunological level, participants displayed typical features of age-associated immune remodeling consistent with physiological immunosenescence. Within this general pattern, however, group-specific immune trajectories differentiated those with new plaques from others. Participants who developed new SAP showed a marked expansion of CD8^+^ EM T cells, together with a stronger decline in Tregs and the Treg/Teff ratio. These immune variables emerged as key predictors of new SAP. In multivariable models, CD8^+^ EM cells remained the most robust independent predictor, while classical risk factors such as blood lipids, glucose, and blood pressure showed no predictive value. Importantly, VCAM-1 also emerged as a strong independent predictor at baseline, highlighting the contribution of early endothelial activation to plaque initiation. Together, these findings suggest that subtle immuno-metabolic dysregulation and endothelial stress may precede overt cardio-metabolic abnormalities in the development of vascular aging.

At baseline, participants were older but generally healthy, with a mean age of 63.8 years and an initial plaque prevalence of 32%. The relatively low prevalence compared to population-based cohorts of similar age [26] supports the interpretation that this cohort represents a healthy aging population, making it particularly suitable for exploring mechanisms of vascular aging. Across all groups, the decline in the CD4^+^/CD8^+^ ratio, loss of naïve CD4, CD8 and CD4^+^ CM T cells, and accumulation of CD4^+^ EM and CD8^+^CD57^+^T cells confirmed typical immunosenescent remodeling known from other studies [27, 28]. Interestingly, within this shared background, individuals who developed new plaques demonstrated distinct shifts in T cell composition, suggesting that vascular aging is shaped not by general immune decline alone but by specific, directional changes within the T cell compartment.

Participants with new SAP displayed a pronounced expansion of CD8^+^ EM T cells during follow-up, whereas these cells remained largely stable in no SAP individuals. CD8^+^ EM T cells are highly differentiated, pro-inflammatory lymphocytes that increase with age and exhibit potent cytotoxic capabilities [29]. Experimental evidence indicates that CD8^+^ EM cells preferentially accumulate within atherosclerotic lesions [30]. Their increase in circulation may reflect systemic immune activation associated with metabolic or endothelial stress [31, 32]. In contrast, CD8^+^ EMRA cells showed distinct trajectories across groups. Their frequencies increased in persistent SAP and no SAP participants but remained stable in the new SAP group. This pattern supports a phase-dependent immune dynamic in which incident plaque formation is associated with an expansion of circulating EM cells, whereas chronic or age-related inflammation favors EMRA accumulation and a more senescent immune phenotype [33].

The marked decline in total Tregs and the Treg/Teff ratio was accompanied by a pronounced shift within the Treg compartment, characterized by a decline in the relative proportion of rTregs and a relative enrichment of mTregs within the remaining Treg population. Thus, the reduction in the total Treg compartment did not reflect a uniform decline across all Treg subsets but was accompanied by distinct subtype-specific trajectories. Although these longitudinal changes were not significantly associated with baseline age and therefore cannot be interpreted as a direct age effect, the observed pattern is consistent with age-associated remodeling of immune regulation described in previous studies [34, 35]. The decline in the Treg/Teff ratio, which was most pronounced in individuals who developed new SAP, indicates a relative shift toward effector-cell dominance and may reflect reduced immune-regulatory capacity during early plaque formation. Tregs are known to limit atherogenesis by suppressing excessive immune activation through cytokines such as IL-10, IL-35, and TGF-β [36]. Thus, the decline in total Tregs relative to Teff cells, together with the subtype-specific shift from rTregs toward an effector-memory-like Treg phenotype, superimposed on broader immunosenescent remodeling, may contribute to an environment that favors endothelial activation and plaque initiation [37]. The concurrent rise in CD8⁺ EM cells and decline in total Tregs and the Treg/Teff ratio, accompanied by distinct changes in rTreg and mTreg proportions, likely reflect impaired regulatory feedback within the adaptive immune network, enabling sustained effector activation and potentially contributing to pro-inflammatory conditions associated with plaque formation. In contrast, participants with persistent SAP did not display a consistent immune pattern that clearly distinguished them from both the no SAP and new SAP groups. SAP are often biologically stable and may remain immunologically quiescent for prolonged periods, during which systemic immune activation is minimal despite the presence of structural disease [10]. This pattern suggests that dynamic immune remodeling is most relevant during the initiation of new plaques rather than the maintenance of established lesions, thereby supporting our focus on predictors of new SAP.

At baseline, immune phenotypes already appeared to predict distinct trajectories of vascular aging. A lower proportion of CD8^+^ EM cells predicted higher odds of future plaque development, whereas elevated CD8^+^ EMRA and Treg frequencies were associated with increased risk. This opposing pattern indicates that baseline circulating CD8^+^ EM frequencies and their longitudinal expansion may capture different stages of immune dysregulation during plaque development. Lower circulating EM frequencies at baseline could reflect several processes, including differences in differentiation, survival, proliferation, or redistribution between the circulation and peripheral tissues. Preferential recruitment to vascular or lymphoid tissues represents one possible explanation; however, cellular trafficking was not directly assessed in the present study and this interpretation therefore remains speculative [30–32]. Longitudinally, an increase in circulating CD8^+^ EM cells emerged as the most consistently associated independent variable. This may indicate that changes in this subset are stage-dependent, with relatively stable baseline levels reflecting immune homeostasis, whereas subsequent expansion may be associated with sustained antigenic or metabolic stimulation, for example in the context of adipose inflammation or endothelial injury. Such persistent stimulation has been linked to activation and eventual exhaustion of CD8^+^ EM cells [38]. Overall, the findings suggest a transition from an initially altered circulating EM profile to a later systemic expansion. However, the underlying mechanisms cannot be determined from the present data. A similar biphasic pattern was observed for the overall Treg compartment. Elevated baseline Treg frequencies were associated with higher plaque risk, whereas a longitudinal decline paralleled disease progression. Within the Treg compartment, however, rTregs declined whereas mTregs became relatively enriched, indicating subtype-specific remodeling rather than a uniform loss of all Treg populations. This may reflect a transition from compensatory to decompensated regulation. Initially, high Treg levels could represent reactive upregulation in response to subclinical inflammatory stress, signaling that an immune system seems to be over-stimulated [17, 39]. Over time, however, chronic activation and cellular senescence may erode the total regulatory pool, particularly its resting/naïve component, thereby reducing control over effector activity [35]. The declining Treg/Teff ratio thus likely marks the failure of an initially compensatory mechanism rather than a simple linear decline. These findings illustrate that immune markers can shift from protective to risk depending on timing, context, and the integrity of regulatory feedback. Early adaptive immune competence manifested as balanced EM and Treg responses may support vascular stability, whereas persistent stimulation and metabolic stress can transform the same cellular programs into drivers of chronic activation and vascular injury.

Multivariable analyses confirmed the independent relevance of immune parameters in predicting plaque formation. Lower CD8^+^ EM cell frequencies remained significantly associated with higher plaque risk after adjustment for age, adiposity, blood pressure, insulin resistance, and physical activity. The initially positive association between CD8^+^ EMRA cells and plaque incidence attenuated after adjustment, indicating that metabolic, fitness and hemodynamic factors partly mediate the link between immune differentiation and vascular injury [40]. Baseline Treg frequencies also lost statistical significance in the fully adjusted model, supporting the notion that regulatory impairment interacts with, rather than acts independently of, metabolic dysregulation [17]. This interdependence highlights that immune and metabolic disturbances are not parallel processes but mutually reinforcing components of vascular aging. Of particular note, VCAM-1 emerged as a strong independent predictor of plaque formation in multivariable models at baseline, although it showed no significant association in univariate analysis. This shift suggests that VCAM-1 reflects vascular risk only when interpreted within the broader metabolic context. VCAM-1 represents endothelial activation and leukocyte adhesion, processes driven by metabolic stress, dyslipidemia, and immune cell trafficking [41, 42]. Its robust association after adjustment indicates that it functions as an integrative biomarker of endothelial stress resulting from immune–metabolic interaction [43]. Mechanistically, this could imply that endothelial dysfunction, captured by rising VCAM-1 levels, represents the vascular manifestation of systemic immune activation rather than an isolated process. Thus, elevated VCAM-1 at baseline may link endothelial activation with the observed circulating immune profile. However, the present data do not establish vascular recruitment or immune-cell redistribution.

Metabolic and functional trajectories closely paralleled the observed immune changes. Participants who developed new SAP showed a progressive increase in visceral adiposity accompanied by a decline in cardiorespiratory fitness over the observation period. These adverse metabolic and functional trajectories were associated with markedly increased odds of incident SAP, underscoring their predictive value for new plaque formation [44]. Expanded visceral fat represents a pro-inflammatory tissue that attracts macrophages and T cells, releasing cytokines such as IFN-γ and TNF-α, which upregulate endothelial adhesion molecules [22, 40]. Adipokines such as leptin further inhibit Treg differentiation and enhance effector activation, linking adiposity directly to immune imbalance [45]. In contrast, higher fitness is associated with lower inflammation and reduced immunosenescence [22, 46]. Declining VO_2_peak likely indicates both reduced anti-inflammatory capacity and lower endothelial shear stress, factors that favor vascular dysfunction [22, 47]. However, neither decrease of VO_2_peak nor increase in visceral fat demonstrated consistent robustness in the multivariable models. This likely reflects the relatively healthy metabolic profile and limited variability within the cohort, which reduced the predictive strength of fitness and adiposity markers. Associations between longitudinal changes in physical fitness, body composition, and immune-cell remodeling in this cohort have been characterized previously [48]. Additional exploratory correlations within the present prediction cohort did not reveal a coherent cross-domain pattern beyond the associations identified in the plaque-focused regression analyses. In contrast, the immune parameters, particularly CD8^+^ EM cells, were more consistently associated with incident SAP and therefore emerged as stronger independent predictors. Despite clear cellular changes, circulating cytokines showed little variation. Neither pro- nor anti-inflammatory mediators displayed consistent group differences or time interactions. This supports the interpretation that cellular immune remodeling may precede measurable shifts in systemic inflammatory markers during subclinical disease stages [49, 50]. Modest increases in GDF-15 and OPG likely represent compensatory or protective responses rather than causal drivers of new SAP. Thus, longitudinal changes in T cell subsets appear to be potentially more sensitive circulating correlates of incident SAP in this cohort than traditional serum cytokines.

A major strength of this study is its longitudinal design, combining immunological, metabolic, and functional measures in a well-characterized, disease-free cohort. This multidimensional approach allowed the identification of circulating immune signatures associated with incident subclinical plaque formation and the examination of dynamic rather than static processes in vascular aging. Several limitations should also be noted. First, the observational design precludes causal inference. Although the three-year follow-up provides temporal information, the exact timing of plaque formation remains unknown, making it difficult to determine whether immune or metabolic changes occur before or after plaque initiation. Second, plaque presence was assessed as a binary outcome without considering number, size, or composition, which limits conclusions about disease extent or progression. Third, immune analyses were restricted to circulating cells and may not fully reflect tissue-resident or plaque-infiltrating immunity. The absence of younger and middle-aged comparison groups further limits our ability to distinguish plaque-associated immune changes from general age-associated immune remodeling. Moreover, plaque-related antigen-specific T cells were not assessed, and cellular trafficking and tissue recruitment were not directly assessed. Therefore, the observed immune signatures cannot be attributed specifically to plaque-related processes, and interpretations involving redistribution of circulating CD8^+^ EM cells remain speculative. Fourth, unmeasured factors such as latent infections, medication use, or lifestyle changes may confound associations despite comprehensive metabolic and functional assessment. Fifth, the moderate sample size may have reduced power to detect smaller effects or complex interactions. Sex and CMV status were explored as additional covariates but were not retained in the final models, most likely due to the small number of female participants and limited statistical power for subgroup analyses. Nevertheless, the internal consistency across immune, metabolic, and functional domains supports the robustness of these findings.

In conclusion, this longitudinal study shows that incident SAP formation in healthy older adults is closely linked to dynamic immune remodeling, even within a metabolically favorable and physically fit cohort. Despite frequent new SAP formation over three-years, classical cardio-metabolic risk markers showed less consistent predictive value. Instead, specific circulating T cell phenotypes and endothelial activation were associated with vascular vulnerability during the subclinical phase of plaque development. Notably, CD8^+^ EM T cells displayed a phase-dependent association with SAP, with lower baseline levels being associated with higher odds of future plaque development and their subsequent expansion representing the strongest independent predictor of new SAP. A similar, albeit less robust, phase-dependent pattern was also observed for the overall Treg compartment. Consistent with this, declining total Treg frequencies and a reduced Treg/Teff ratio indicated a tilt toward effector dominance, whereas the reduction in rTregs and relative enrichment of mTregs reflected subtype-specific remodeling within the remaining Treg compartment. The strong association of VCAM-1 with plaque formation supports a role for endothelial activation within this systemic immune–metabolic profile. Importantly, participants who developed new SAP were characterized by a progressive increase in total and visceral adiposity together with a decline in cardiorespiratory fitness, and these adverse metabolic and functional trajectories increased the odds of incident SAP. Integrating immune, metabolic, and functional trajectories supports an immuno-metabolic model of vascular aging, in which subtle immune dysregulation, increasing adiposity, and declining fitness are jointly associated with the subclinical phase of plaque development. These findings highlight adaptive immune signatures, particularly CD8^+^ EM dynamics, Treg remodeling, and VCAM-1 as central components associated with incident SAP in healthy aging and suggest that longitudinal immune profiling combined with preservation of metabolic and functional health may inform future risk-stratification and preventive strategies, pending validation in larger independent cohorts.

## Supplementary Information

Supplementary Material 1.

## Data Availability

No datasets were generated or analysed during the current study.
