# Supplementary material for: CD8+ effector memory and regulatory T cell dynamics predict subclinical atherosclerotic plaque formation in healthy aging
Source: Immun Ageing. 2026 Aug 15;23:29. doi: 10.1186/s12979-026-00588-2 (PMC13499307; doi:10.1186/s12979-026-00588-2)
Supplement: Supplementary file 1 [file 12979_2026_588_MOESM1_ESM.docx]

**Figure S1**. Frozen PBMCs were thawed and stained with fluorochrome-conjugated antibodies. Gating strategy: Lymphocytes were gated in a FSC/SSC-dot plot and dead cells excluded using Zombie Aqua^TM^. From the living cell population, CD4^+^ and CD8^+^T-cells as well as CD4^-^CD8^-^ lymphocytes, were gated according. T cell subsets were gated in CD4+ and CD8+ T cells as naïve T cells (CCR7^+^CD45RA^+^), Central Memory T cells (CCR7^+^CD45RA^-^), Effector Memory T cells (CCR7^-^CD45RA^-^) and effector memory T cells re-expressing CD45RA (EMRA) (CCR7^-^CD45RA^+^). Additionally, CD57^+^ were gated on CD4^+^ and CD8^+^ T cells as CD4^+^CD57^+^ and CD8^+^CD57^+^.

**Figure S2**. Frozen PBMCs were thawed and stained with fluorochrome-conjugated antibodies. Gating strategy: Lymphocytes were gated in a FSC/SSC-dot plot and dead cells excluded using Zombie Aqua^TM^. From the living cell population, CD4 T cells, were gated according to CD3^+^/CD4^+^ (A). T effector cells (Teff) were gated as CD4^+^CD25^+^CD127^high^. T_regs_ were defined and gated as CD4^+^CD25^+^CD127^low^. Subsets of Tregs were gated as resting/ naïve Tregs (rTregs) (CD45RO^-^CCR7^+^) and effector-memory-like Tregs (mTregs) (CD45RO^+^CCR7^-^).

**Figure S3.** Representative flow cytometry plots of CD8⁺ T-cell differentiation (A) and regulatory T-cell identification (B). (A) Representative plots illustrating the distribution of naïve, central memory (CM), effector memory (EM), and terminally differentiated effector memory RA-positive (EMRA) CD8⁺ T cells based on CCR7 and CD45RA expression. (B) Representative plots showing the identification of regulatory T cells (Tregs) and conventional effector T cells (Teff) based on CD25 and CD127 expression. Plots are shown for participants without SAP and participants who developed new SAP at baseline and follow-up. Panel A is based on live singlet CD8⁺ T cells, whereas panel B is based on live singlet CD3⁺CD4⁺ T cells. The corresponding preceding gating steps are not shown.

**Table S1.** Longitudinal changes of anthropometrics in healthy older adults over a 3-year follow-up according to no subclinical atherosclerotic plaque (SAP), new SAP and persistent SAP status.

|  | No SAP | | New SAP | | Persistent SAP | | Time  p-value | Time x group  p-value |
| --- | --- | --- | --- | --- | --- | --- | --- | --- |
|  | **Baseline** | **Follow-up** | **Baseline** | **Follow-up** | **Baseline** | **Follow-up** |  |  |
| BMI (kg/m²) | 24.2 ± 2.7 | 24.0 ± 2.7 | 26.6 ± 4.0 | 26.6 ± 3.3 | 25.1 ± 3.1 | 24.7 ± 3.4 | 0.257 | 0.672 |
| Body Fat % | 28.8 ± 6.3 | 28.8 ± 6.6 | 27.6 ± 9.1 | 30.7 ± 7.0 | 26.3 ± 6.6 | 26.5 ± 6.8 | **0.016^b^** | **0.019^#^** |
| Visceral Fat % | 31.3 ± 5.1 | 31.1 ± 5.3 | 31.4 ± 8.4 | 34.9 ± 5.3 | 29.4 ± 6.2 | 29.8 ± 6.7 | **0.018^b^** | **0.020^#^** |
| Hand grip (kg) | 43.3 ± 8.6 | 37.4 ± 8.6 | 42.9 ± 9.6 | 36.8 ± 8.4 | 46.6 ± 12.1 | 40.7 ± 14.6 | **≤0.001^a,b,c^** | 0.994 |
| VO_2_peak (ml/min/kg) | 30.7 ± 6.6 | 30.7 ± 7.0 | 28.9 ± 7.7 | 25.2 ± 5.6 | 31.4 ± 7.7 | 28.3 ± 7.0 | **≤0.001^c^** | **0.012^#,§^** |
| METs min/week | 5703.6 ± 3074.0 | 6311.8 ± 3061.6 | 3594.6 ± 2085.1 | 2582.4 ± 2046.6 | 6060.6 ± 3445.6 | 6298.4 ± 4481.0 | 0.907 | 0.448 |
| Systolic BP (mmHg) | 123.5 ± 9.0 | 127.1 ± 9.9 | 121.5 ± 9.2 | 125.7 ± 11.8 | 130.8 ± 11.4 | 133.3 ± 13.9 | 0.054 | 0.919 |
| Diastolic BP (mmHg) | 75.2 ± 10.0 | 77.9 ± 8.5 | 71.4 ± 8.5 | 72.7 ± 9.1 | 76.3 ± 8.6 | 76.8 ± 8.6 | 0.232 | 0.704 |

^a^ indicates post-hoc differences within No SAP Group, ^b^ indicates post-hoc differences within New SAP Group, ^c^ indicates post-hoc differences within Persistent SAP Group. # indicates differences between no SAP and new SAP, § differences between no SAP and persistent SAP, ǂ differences between new SAP and persistent SAP. **Abbreviations:** CD: cluster of differentiation; CM: central memory T cells; EM: effector memory T cells; EMRA: effector memory T cells re-expressing CD45RA; Teff: T effector cells; Treg: regulatory T cells; rTreg: naïve Treg cells; mTreg: memory Treg cells.

**Table S2.** Longitudinal changes in metabolic and endocrine parameters according to subclinical atherosclerotic plaque status

|  | No SAP | | New SAP | | Persistent SAP | | Time | Time x group  p-value |
| --- | --- | --- | --- | --- | --- | --- | --- | --- |
|  | **Baseline** | **Follow-up** | **Baseline** | **Follow-up** | **Baseline** | **Follow-up** |  |  |
| Glucose (mg/dl) | 98.1 ± 7.9 | 100.3 ± 7.6 | 95.7 ± 8.6 | 97.1 ± 5.6 | 102.3 ± 11.1 | 106.5 ± 12.9 | **0.040** | 0.631 |
| HbA1c (%) | 5.6 ± 0.3 | 5.5 ± 0.3 | 5.5 ± 0.4 | 5.4 ± 0.3 | 5.6 ± 0.4 | 5.6 ± 0.4 | 0.323 | 0.804 |
| Insulin mU/l | 5.3 ± 2.0 | 6.2 ± 3.0 | 6.8 ± 3.1 | 6.1 ± 2.8 | 6.4 ± 3.2 | 7.5 ± 5.0 | 0.413 | 0.267 |
| HOMA-IR | 1.3 ± 0.5 | 1.5 ± 0.8 | 1.6 ± 0.8 | 1.5 ± 0.7 | 1.6 ± 0.9 | 2.0 ±1.5 | 0.279 | 0.342 |
| Total cholesterol (mg/dl) | 227.2 ± 33.8 | 235.3 ± 36.2 | 230.1 ± 35.9 | 237.7 ± 41.4 | 204.1 ± 26.7 | 206.1 ± 34.1 | 0.076 | 0.672 |
| LDL-Cholesterol (mg/dl) | 150.3 ± 32.8 | 163.7 ± 40.8 | 164.1 ±34.2 | 175.6 ± 39.9 | 142.6 ± 32.7 | 146.4 ± 34.3 | **0.027** | 0.563 |
| HDL-Cholesterol (mg/dl) | 62.4 ± 16.1 | 60.8 ±15.3 | 57.1 ± 15.0 | 57.4 ± 14.5 | 60.3 ± 12.2 | 60.6 ± 10.0 | 0.718 | 0.593 |
| LDL/ HDL ratio | 2.57 ± 0.28 | 2.92 ± 1.12 | 3.04 ± 0.90 | 3.22 ± 0.94 | 2.50 ± 0.96 | 2.46 ± 0.93 | **0.019 ^a^** | **0.031^§^** |
| Cortisol (µg/dl) | 16.1 ± 4.3 | 13.7 ± 2.0 | 13.5 ± 2.8 | 12.2 ±3.5 | 15.2 ± 5.2 | 12.9 ± 2.9 | **0.002 ^a^** | 0.743 |

^a^ indicates post-hoc differences within No SAP Group, ^b^ indicates post-hoc differences within New SAP Group, ^c^ indicates post-hoc differences within Persistent SAP Group. # indicates differences between no SAP and new SAP, § differences between no SAP and persistent SAP, ǂ differences between new SAP and persistent SAP. **Abbreviations:** HbA1c: glycated hemoglobin; HOMA-IR: homeostasis model assessment of insulin resistance; LDL: low-density lipoprotein; HDL: high-density lipoprotein.

**Table S3.** Longitudinal changes in circulating T cell subsets according to subclinical atherosclerotic plaque status.

|  | No SAP | | New SAP | | Persistent SAP | | Time | Time x group  p-value |
| --- | --- | --- | --- | --- | --- | --- | --- | --- |
|  | **Baseline** | **Follow-up** | **Baseline** | **Follow-up** | **Baseline** | **Follow-up** |  |  |
| CD4^+^/CD8^+^ ratio | 1.8 ± 0.8 | 1.4 ± 0.8 | 1.9 ± 1.1 | 1.4 ± 1.1 | 2.3 ± 2.1 | 1.5 ± 1.4 | **0.006** | 0.712 |
| CD4^+^ naïve (%) | 37.9 ± 16.5 | 27.4 ± 13.5 | 39.7 ± 17.5 | 28.4 ± 17.6 | 32.2 ± 17.2 | 27.7 ± 18.1 | **≤0.001^a^** | 0.474 |
| CD4^+^ CM (%) | 22.2 ± 8.2 | 30.0 ± 10.1 | 27.9 ± 12.4 | 27.8 ± 11.3 | 29.0 ± 13.6 | 28.4 ± 14.7 | 0.118 | **0.020^§^** |
| CD4^+^ EM (%) | 37.0 ± 17.3 | 39.8 ± 16.2 | 26.3 ± 12.8 | 39.3 ± 14.9 | 30.7 ± 16.1 | 36.6 ± 17.2 | **0.022** | 0.415 |
| CD4^+^ EMRA (%) | 2.9 ± 2.2 | 2.5 ± 3.2 | 6.1 ± 9.3 | 4.6 ± 5.6 | 8.1 ± 10.7 | 6.2 ±9.7 | 0.083 | 0.596 |
| CD4^+^CD57^+^ (%) | 8.6 ± 13.4 | 9.7 ± 12.8 | 7.9 ± 11.2 | 5.7 ± 7.8 | 9.9 ± 14.3 | 10.8 ± 18.4 | 0.952 | 0.604 |
| CD8^+^ naïve (%) | 14.5 ± 16.4 | 7.0 ± 5.1 | 14.6 ± 16.5 | 4.2 ± 4.4 | 14.7 ± 15.9 | 5.9 ± 5.1 | **≤0.001^a,c^** | 0.865 |
| CD8^+^ CM (%) | 5.4 ± 6.2 | 3.0 ± 3.1 | 4.1 ± 3.3 | 2.2 ±1.9 | 5.9 ± 8.4 | 3.1 ± 2.7 | **0.006^a,b^** | 0.919 |
| CD8^+^ EM (%) | 38.2 ± 13.6 | 36.3 ± 14.0 | 24.4 ± 12.3 | 42.6 ±17.8 | 28.0 ± 16.2 | 34.5 ± 12.6 | **0.008^b^** | **0.014^#^** |
| CD8^+^ EMRA (%) | 41.8 ±15.6 | 53.4 ± 14.6 | 57.0 ± 18.6 | 51.1 ± 18.0 | 39.8 ± 20.0 | 55.7 ± 12.5 | **0.015^a,c^** | **0.017^§,^**^ǂ^ |
| CD8^+^CD57^+^ (%) | 37.7 ± 14.9 | 46.1 ± 15.4 | 44.5 ± 16.1 | 52.3 ± 13.1 | 40.3 ± 13.4 | 42.6 ±15.9 | **0.002^a^** | 0.605 |
| Teff (%) | 74.3 ± 7.5 | 79.3 ± 12.1 | 77.6 ± 5.0 | 87.4 ± 5.5 | 76.8 ± 7.1 | 84.0 ± 8.5 | **≤0.001^a,b,c^** | 0.332 |
| Treg (%) | 5.7 ± 1.5 | 2.9 ± 1.4 | 7.1 ± 1.8 | 2.6 ± 1.3 | 5.6 ±1.3 | 3.3 ± 1.8 | **≤0.001^a,b,c^** | **0.023^#,^**^ǂ^ |
| Treg/Teff Ratio | 0.08 ± 0.02 | 0.04 ± 0.02 | 0.10 ± 0.02 | 0.03 ± 0.01 | 0.08 ±0.02 | 0.04 ± 0.03 | **≤0.001^a,b,c^** | **0.003^#,^**^ǂ^ |
| rTreg (%) | 36.0 ± 11.4 | 9.6 ± 11.6 | 39.7 ± 17.5 | 11.6 ± 7.0 | 28.0 ± 9.4 | 10.0 ± 8.6 | **≤0.001^a,b,c^** | 0.062 |
| mTreg (%) | 25.9 ± 10.1 | 57.9 ± 21.7 | 27.9 ± 12.4 | 48.2 ± 18.2 | 38.8 ± 13.8 | 58.2 ± 19.0 | **≤0.001^a,b,c^** | 0.112 |

CD4⁺ and CD8⁺ subsets are reported as percentages of their respective parent populations. Treg and Teff frequencies are reported as percentages of total CD3⁺CD4⁺ T cells; rTreg and mTreg frequencies are reported as percentages of total Tregs. ^a^ indicates post-hoc differences within No SAP Group, ^b^ indicates post-hoc differences within New SAP Group, ^c^ indicates post-hoc differences within Persistent SAP Group. # indicates differences between no SAP and new SAP, § differences between no SAP and persistent SAP, ǂ differences between new SAP and persistent SAP. **Abbreviations:** CD: cluster of differentiation; CM: central memory T cells; EM: effector memory T cells; EMRA: effector memory T cells re-expressing CD45RA; Teff: T effector cells; Treg: regulatory T cells; rTreg: naïve Treg cells; mTreg: memory Treg cells.

**Table S4.** Longitudinal changes in circulating inflammatory and vascular biomarkers according to subclinical atherosclerotic plaque status

|  | No SAP | | New SAP | | Persistent SAP | | Time | Time x group  p-value |
| --- | --- | --- | --- | --- | --- | --- | --- | --- |
|  | **Baseline** | **Follow-up** | **Baseline** | **Follow-up** | **Baseline** | **Follow-up** |  |  |
| CCL-2 (pg/ml) | 497.9 ± 149.0 | 488.2 ± 141.5 | 466.7 ± 155.2 | 438.1 ± 162.6 | 513.5 ± 173.8 | 518.2 ± 172.7 | 0.469 | 0.716 |
| CXCL-9 (pg/ml) | 945.8 ± 1501.8 | 871.5 ± 1290.7 | 863.0 ± 1519.6 | 1117.1 ±2092.5 | 746.1 ± 962.2 | 640.9 ± 787.3 | 0.698 | 0.083 |
| CXCL-10 (pg/ml) | 11.0 ± 4.5 | 11.2 ± 5.6 | 13.9 ± 12.1 | 14.0 ± 14.3 | 12.8 ± 7.1 | 12.1 ± 6.5 | 0.837 | 0.695 |
| FGF-23 (pg/ml) | 37.8 ± 107.1 | 37.2 ±124.7 | 158.9 ± 475.5 | 312.2 ± 956.0 | 6.6 ± 2.6 | 6.0 ± 2.2 | 0.123 | 0.138 |
| GDF-15 (pg/ml) | 522.6 ± 207.6 | 578.2 ± 193.2 | 590.7 ± 348.8 | 669.8 ± 473.3 | 634.6 ± 403.4 | 667.6 ± 431.0 | **0.001^a^** | 0.567 |
| IL-1ra (pg/ml) | 822.0 ± 1196.5 | 901.0 ± 1388.0 | 996.1 ± 1791.4 | 1099.7 ± 1765.3 | 421.1 ± 132.1 | 537.0 ± 315.1 | **0.022** | 0.916 |
| IL-6 (pg/ml) | 15.8 ± 29.2 | 16.4 ± 30.4 | 34.1 ± 71.6 | 50.1 ± 119.1 | 14.6 ± 14.2 | 21.6 ± 38.4 | 0.073 | 0.350 |
| IL-10 (pg/ml) | 1.7 ± 1.4 | 1.6 ± 1.4 | 2.4 ± 1.5 | 2.6 ± 1.7 | 1.7 ± 1.2 | 1.7 ± 1.5 | 0.520 | 0.646 |
| IL-18 (pg/ml) | 473.2 ± 278.8 | 484.6 ± 310.3 | 390.4 ± 173.3 | 390.5 ± 195.6 | 613.9 ± 278.7 | 609.6 ± 316.6 | 0.882 | 0.892 |
| Osteopontin (pg/ml) | 24424.9 ± 13772.3 | 21011.3 ± 15678.8 | 30980.1 ± 22480.6 | 24128.1 ± 14986.6 | 48489.7 ± 32719.2 | 37399.2 ± 31630.1 | 0.053 | 0.613 |
| Osteoprotegerin (pg/ml) | 919.3 ± 402.8 | 929.8 ± 467.3 | 1285.2 ± 1551.9 | 1453.2 ± 1987.7 | 752.4 ± 199.7 | 843.4 ± 266.2 | **0.014^c^** | 0.192 |
| TNF-α (pg/ml) | 6.3 ± 6.4 | 6.0 ± 5.5 | 7.5 ± 5.1 | 9.1 ± 9.5 | 6.0 ± 4.0 | 5.9 ± 2.7 | 0.343 | 0.319 |
| TRAIL (pg/ml) | 43.1 ± 25.3 | 42.0 ± 23.9 | 56.3 ± 31.9 | 59.4 ± 43.0 | 37.6 ±13.6 | 35.4 ± 14.7 | 0.987 | 0.718 |
| VCAM-1 (pg/ml) | 946 991.1 ± 348 457.4 | 971 035.2 ± 376 362.0 | 1 162 000.0 ± 375 301.2 | 1 178 000.0 ± 404 469.7 | 994 557.9 ± 494 225.5 | 1 064 000.0 ± 521 442.8 | 0.256 | 0.751 |
| VEGF (pg/ml) | 501.5 ± 446.0 | 518.4 ± 385.2 | 410.4 ± 438.3 | 481.1 ± 527.9 | 617.9 ± 819.5 | 483.6 ± 369.0 | 0.824 | 0.471 |

^a^ indicates post-hoc differences within No SAP Group, ^b^ indicates post-hoc differences within New SAP Group, ^c^ indicates post-hoc differences within Persistent SAP Group. # indicates differences between no SAP and new SAP, § differences between no SAP and persistent SAP, ǂ differences between new SAP and persistent SAP. **Abbreviations:** CCL: chemokine (C-C motif) ligand; CXCL: chemokine (C-X-C motif) ligand; FGF: fibroblast growth factor; GDF: growth differentiation factor; IL: interleukin; TNF: tumor necrosis factor; VCAM: vascular cell adhesion molecule; VEGF: vascular endothelial growth factor

**Table S5** Associations between baseline measures in clinical, metabolic, and immunological parameters and subclinical atherosclerotic plaque progression

| Parameter | Odds Ratio | (95% CI) | p-value |
| --- | --- | --- | --- |
| Age | 1.17 | 0.95 – 1.45 | 0.145 |
| Sex (w) | 0.47 | 0.08 – 2.76 | 0.402 |
| BMI (kg/m²) | 1.27 | 0.98 – 1.65 | 0.067 |
| Body fat (%) | 0.98 | 0.88 – 1.09 | 0.654 |
| Visceral fat (%) | 1.00 | 0.89 – 1.13 | 0.968 |
| Hand grip strength (kg) | 1.01 | 0.92 – 1.10 | 0.888 |
| VO_2_peak (ml/min/kg) | 0.96 | 0.86 – 1.08 | 0.495 |
| MET’s (min/week) | 0.37 | 0.11 – 1.23 | 0.185 |
| Glucose (mg/dl) | 0.96 | 0.87 – 1.06 | 0.645 |
| Insulin (mU/l) | 1.29 | 0.93 – 1.79 | 0.157 |
| HOMA-IR | 2.49 | 0.70 – 8.82 | 0.121 |
| HbA1c (%) | 0.43 | 0.03 – 5.38 | 0.510 |
| Total cholesterol (mg/dl) | 1.00 | 0.98 – 1.03 | 0.820 |
| LDL-Cholesterol (mg/dl) | 1.01 | 0.99 – 1.04 | 0.274 |
| HDL-Cholesterol (mg/dl) | 0.98 | 0.93 – 1.03 | 0.372 |
| LDL/HDL-ratio | 2.00 | 0.77 – 5.16 | 0.153 |
| Cortisol (µg/dl) | 0.83 | 0.66 – 1.04 | 0.108 |
| CMV (positive) | 0.83 | 0.19 – 3.72 | 0.811 |
| Systolic BP (mmHg) | 0.97 | 0.89 – 1.06 | 0.546 |
| Diastolic BP (mmHg) | 0.96 | 0.88 – 1.04 | 0.300 |
| T cells | **Odds Ratio** | **95% CI** | **p-value** |
| CD4^+^/CD8^+^ ratio | 1.15 | 0.49 – 2.72 | 0.750 |
| CD4^+^ naive (%) | 1.01 | 0.96 – 1.05 | 0.770 |
| CD4^+^ CM (%) | 1.06 | 0.98 – 1.15 | 0.135 |
| CD4^+^ EM (%) | 0.95 | 0.89 – 1.01 | 0.101 |
| CD4^+^ EMRA (%) | 1.13 | 0.93 – 1.38 | 0.232 |
| CD4^+^CD57^+^ (%) | 1.00 | 0.94 – 1.06 | 0.883 |
| CD8^+^ naive (%) | 1.00 | 0.95 – 1.05 | 0.999 |
| CD8^+^ CM (%) | 0.94 | 0.79 – 1.13 | 0.512 |
| CD8^+^ EM (%) | 0.92 | 0.86 – 0.99 | **0.022** |
| CD8^+^ EMRA (%) | 1.06 | 1.01 – 1.12 | **0.033** |
| CD8^+^CD57^+^ (%) | 1.03 | 0.98 – 1.09 | 0.248 |
| Teff (%) | 1.09 | 0.95 – 1.24 | 0.217 |
| Treg (%) | 1.67 | 1.01 – 2.76 | **0.045** |
| Treg/Teff ratio | 1.66 | 0.78 – 3.52 | 0.186 |
| rTreg (%) | 0.97 | 0.90 – 1.05 | 0.437 |
| mTreg (%) | 1.05 | 0.98 – 1.13 | 0.151 |
| Serum Proteins | **Odds Ratio** | **95% CI** | **p-value** |
| CCL-2 (pg/ml) | 0.80 | 0.36 – 1.76 | 0.577 |
| CXCL-9 (pg/ml) | 0.95 | 0.47 – 1.90 | 0.881 |
| CXCL-10 (pg/ml) | 1.42 | 0.69 – 2.93 | 0.344 |
| FGF-23 (pg/ml) | 1.43 | 0.71 – 2.90 | 0.319 |
| GDF-15 (pg/ml) | 1.38 | 0.57 – 3.38 | 0.479 |
| IL-1ra (pg/ml) | 1.11 | 0.62 – 2.00 | 0.736 |
| IL-6 (pg/ml) | 1.36 | 0.74 – 2.52 | 0.321 |
| IL-10 (pg/ml) | 1.60 | 0.79 – 3.24 | 0.193 |
| IL-18 (pg/ml) | 0.65 | 0.24 – 1.74 | 0.387 |
| Osteopontin (pg/ml) | 1.81 | 0.58 – 5.60 | 0.305 |
| Osteoprotegerin (pg/ml) | 1.38 | 0.72 – 2.65 | 0.338 |
| TNF-α (pg/ml) | 1.20 | 0.63 – 2.25 | 0.580 |
| TRAIL (pg/ml) | 1.51 | 0.78 – 2.92 | 0.217 |
| VCAM-1 (pg/ml) | 1.96 | 0.83 – 3.98 | 0.126 |
| VEGF (pg/ml) | 0.73 | 0.23 – 2.25 | 0.583 |

Serum protein concentrations and physical activity values were log-transformed and standardized before logistic regression analysis. Corresponding odds ratios are reported per one-standard-deviation increase in the log-transformed variable. **Abbreviations:** CCL: chemokine (C-C motif) ligand CD: cluster of differentiation; CI: confidence interval; CM: central memory T cells; CXCL: chemokine (C-X-C motif) ligand EM: effector memory T cells; EMRA: effector memory T cells re-expressing CD45RA; FGF: fibroblast growth factor; GDF: growth differentiation factor; HbA1c: glycated hemoglobin; HOMA-IR: homeostasis model assessment of insulin resistance; HDL: high-density lipoprotein.IL: interleukin; LDL: low-density lipoprotein; TNF: tumor necrosis factor;Teff: T effector cells; Treg: regulatory T cells; rTreg: naïve Treg cells; mTreg: memory Treg cells; VCAM: vascular cell adhesion molecule; VEGF: vascular endothelial growth factor

**Table S6** Associations between longitudinal changes in clinical, metabolic, and immunological parameters and subclinical atherosclerotic plaque progression

| Parameter | **Odds Ratio** | **95% CI** | **p-value** |
| --- | --- | --- | --- |
|  |  |  |  |
| Age (baseline) | 1.17 | 0.95 – 1.45 | 0.145 |
| ΔBMI (kg/m²) | 1.33 | 0.65 – 2.71 | 0.435 |
| ΔBody fat (%) | 1.47 | 1.06 – 2.04 | **0.023** |
| ΔVisceral fat (%) | 1.51 | 1.07 – 2.13 | **0.018** |
| ΔHand grip strength (kg) | 0.98 | 0.76 – 1.27 | 0.897 |
| ΔVO_2_peak (ml/min/kg) | 0.73 | 0.54 – 0.98 | **0.035** |
| ΔMET’s (min/week) | 0.38 | 0.08 – 1.72 | 0.208 |
| Δglucose (mg/dl) | 0.99 | 0.89 – 1.09 | 0.798 |
| ΔInsulin (mU/l) | 0.72 | 0.47 – 1.10 | 0.125 |
| ΔHOMA-IR | 0.33 | 0.07 – 1.54 | 0.158 |
| ΔHbA1c (%) | 0.85 | 0.34 – 2.12 | 0.733 |
| Δtotal cholesterol (mg/dl) | 1.00 | 0.96 – 1.04 | 0.955 |
| ΔLDL-Cholesterol (mg/dl) | 1.00 | 0.97 – 1.03 | 0.849 |
| ΔHDL-Cholesterol (mg/dl) | 1.06 | 0.93 – 1.21 | 0.392 |
| ΔLDL/HDL-ratio | 0.46 | 0.09 – 2.45 | 0.362 |
| ΔCortisol (µg/dl) | 1.10 | 0.88 – 1.38 | 0.389 |
| ΔSystolic BP (mmHg) | 1.00 | 0.94 – 1.07 | 0.879 |
| ΔDiastolic BP (mmHg) | 0.98 | 0.90 – 1.07 | 0.665 |
| T cells | **Odds Ratio** | **95% CI** | **p-value** |
| ΔCD4^+^/CD8^+^ ratio | 0.93 | 0.43 – 2.00 | 0.850 |
| ΔCD4^+^ naive (%) | 1.00 | 0.95 – 1.04 | 0.897 |
| ΔCD4^+^ CM (%) | 0.92 | 0.85 – 1.00 | 0.054 |
| ΔCD4^+^ EM (%) | 1.03 | 0.98 – 1.07 | 0.182 |
| ΔCD4^+^ EMRA (%) | 0.90 | 0.70 – 1.14 | 0.380 |
| ΔCD4^+^CD57^+^ (%) | 0.97 | 0.89 – 1.05 | 0.398 |
| ΔCD8^+^ naive (%) | 0.99 | 0.94 – 1.04 | 0.610 |
| ΔCD8^+^ CM (%) | 1.03 | 0.87 – 1.23 | 0.746 |
| ΔCD8^+^ EM (%) | 1.08 | 1.01 – 1.15 | **0.027** |
| ΔCD8^+^ EMRA (%) | 0.93 | 0.87 – 1.00 | 0.061 |
| ΔCD8^+^CD57^+^ (%) | 1.00 | 0.94 – 1.06 | 0.920 |
| ΔTeff (%) | 1.09 | 0.96 – 1.24 | 0.181 |
| ΔTreg (%) | 0.57 | 0.34 – 0.95 | **0.031** |
| ΔTreg/Teff ratio | 0.19 | 0.05 – 0.67 | **0.010** |
| ΔrTreg (%) | 1.04 | 0.97 – 1.11 | 0.264 |
| ΔmTreg (%) | 0.98 | 0.95 – 1.01 | 0.118 |
| Serum Proteins | **Odds Ratio** | **95% CI** | **p-value** |
| ΔCCL-2 (pg/ml) | 0.83 | 0.40 – 1.74 | 0.622 |
| ΔCXCL-9 (pg/ml) | 2.32 | 0.81 – 5.62 | 0.117 |
| ΔCXCL-10 (pg/ml) | 0.97 | 0.46– 2.05 | 0.935 |
| ΔFGF-23 (pg/ml) | 3.36 | 0.01 – 864.76 | 0.668 |
| ΔGDF-15 (pg/ml) | 1.23 | 0.59 – 2.57 | 0.589 |
| ΔIL-1ra (pg/ml) | 1.09 | 0.52 – 2.29 | 0.822 |
| ΔIL-6 (pg/ml) | 2.17 | 0.37 – 12.56 | 0.388 |
| ΔIL-10 (pg/ml) | 1.45 | 0.68 – 3.12 | 0.338 |
| ΔIL-18 (pg/ml) | 0.88 | 0.42 – 1.84 | 0.737 |
| ΔOsteopontin (pg/ml) | 0.76 | 0.37 – 1.58 | 0.463 |
| ΔOsteoprotegerin (pg/ml) | 1.86 | 0.67 – 5.19 | 0.236 |
| ΔTNF-α (pg/ml) | 1.64 | 0.73 – 3.72 | 0.234 |
| ΔTRAIL (pg/ml) | 1.27 | 0.58 – 2.80 | 0.547 |
| ΔVCAM-1 (pg/ml) | 0.96 | 0.46 – 2.01 | 0.918 |
| ΔVEGF (pg/ml) | 1.38 | 0.65 – 2.96 | 0.405 |

Serum protein concentrations and physical activity values were log-transformed and standardized before logistic regression analysis. Corresponding odds ratios are reported per one-standard-deviation increase in the log-transformed variable. **Abbreviations:** CCL: chemokine (C-C motif) ligand CD: cluster of differentiation; CI: confidence interval; CM: central memory T cells; CXCL: chemokine (C-X-C motif) ligand EM: effector memory T cells; EMRA: effector memory T cells re-expressing CD45RA; FGF: fibroblast growth factor; GDF: growth differentiation factor; HbA1c: glycated hemoglobin; HOMA-IR: homeostasis model assessment of insulin resistance; HDL: high-density lipoprotein.IL: interleukin; LDL: low-density lipoprotein; NF: tumor necrosis factor;Teff: T effector cells; Treg: regulatory T cells; rTreg: naïve Treg cells; mTreg: memory Treg cells; VCAM: vascular cell adhesion molecule; VEGF: vascular endothelial growth factor

|  | Model 1 | Model 2 | Model 3 | Model 4 | Model 5 | Model 6 |
| --- | --- | --- | --- | --- | --- | --- |
|  | OR (95% CI) | OR (95% CI) | OR (95% CI) | OR (95% CI) | OR (95% CI) | OR (95% CI) |
| CD8^+^ EM (%) | 0.92 (0.85 – 0.99)* | 0.92 (0.85 – 0.99)* | 0.91 (0.84 – 0.99)* | 0.91 (0.84 – 0.99)* | 0.92 (0.84 – 0.99)* | 0.92 (0.84 – 0.99)* |
| BMI (kg/m²) | 1.32 (0.94 – 1.87) | 1.26 (0.84 – 1.90) | 1.24 (0.82 – 1.88) | 1.27 (0.82 – 1.96) | 1.22 (0.20 – 3.23) | 1.33 (0.78 – 2.25) |
| HOMA-IR | - | 1.31 (0.21 – 8.16) | 1.41 (0.21 – 9.23) | 1.61 (0.20 – 13.12) | 1.72 (0.21 – 14.00) | 1.54 (0.17 – 14.1) |
| Age | - | - | 1.16 (0.92 – 1.46) | 1.17 (0.92 – 1.49) | 1.17 (0.92 – 1.46) | 1.21 (0.93 – 1.58) |
| LDL/HDL ratio | – | - | - | 0.78 (0.16 – 3.89) | 0.88 (0.17 – 4.95) | 0.97 (0.18 – 5.27) |
| METs (min/week) | – | – | - | - | 0.63 (0.13 – 3.05) | 0.67 (0.11 – 3.98) |
| Systolic BP (mmHg) | – | – | – | - | - | 0.92 (0.79 – 1.08) |
| Χ² | 10.27** | 10.61* | 12.28* | 12.37* | 12.77* | 13.90* |

**Table S7** Multivariable logistic regression models assessing the association of CD8+ effector memory T cells with subclinical atherosclerotic plaque status

**Abbreviations:** CI: confidence interval; OR: odds ratio; BMI: body mass index; HOMA-IR: homeostasis model assessment of insulin resistance; MET: metabolic equivalent of task; BP: blood pressure. CD8+ EM: CD8+ effector memory T cells.

**Table S8** Multivariable logistic regression models assessing the association of CD8+ EMRA T cells with subclinical atherosclerotic plaque status

|  | Model 1 | Model 2 | Model 3 | Model 4 | Model 5 | Model 6 |
| --- | --- | --- | --- | --- | --- | --- |
|  | OR (95% CI) | OR (95% CI) | OR (95% CI) | OR (95% CI) | OR (95% CI) | OR (95% CI) |
| CD8^+^ EMRA (%) | 1.11 (1.01 – 1.12)* | 1.14 (1.02 – 1.27)* | 1.14 (1.02 – 1.29)* | 1.15 (1.01 – 1.30)* | 1.14 (1.01 – 1.30)* | 1.13 (0.99 – 1.29) |
| BMI (kg/m²) | 1.54 (1.06 – 2.24)* | 1.75 (1.05 – 2.92)* | 1.74 (1.01 – 2.98)* | 1.73 (0.99 – 3.00) | 1.61 (0.90 – 2.88) | 1.61 (0.90 – 2.88) |
| HOMA-IR | - | 0.46 (0.06 – 3.41) | 0.43 (0.05 – 3.58) | 0.38 (0.03 – 4.33) | 0.52 (0.04 – 6.81) | 0.53 (0.04 – 7.00) |
| Age | - | - | 1.14 (0.89 – 1.45) | 1.13 (0.89 – 1.45) | 1.14 (0.88 – 1.47) | 1.15 (0.87 – 1.50) |
| LDL/HDL ratio | – | - | - | 1.17 (0.25 – 5.60) | 1.23 (0.26 – 5.89) | 1.23 (0.26 – 5.93) |
| METs (min/week) | – | – | - | - | 0.59 (0.09 – 3.82) | 0.60 (0.09 – 3.93) |
| Systolic BP (mmHg) | – | – | – | - | - | 0.99 (0.84 – 1.17) |
| Χ² | 12.61** | 14.31** | 15.043** | 15.47** | 15.79* | 15.80* |

**Abbreviations:** CI: confidence interval; OR: odds ratio; BMI: body mass index; HOMA-IR: homeostasis model assessment of insulin resistance; MET: metabolic equivalent of task; BP: blood pressure. EMRA: effector memory T cells re-expressing CD45RA.

**Table S9** Multivariable logistic regression models assessing the association of regulatory T cells with subclinical atherosclerotic plaque status

|  | Model 1 | Model 2 | Model 3 | Model 4 | Model 5 | Model 6 |
| --- | --- | --- | --- | --- | --- | --- |
|  | OR (95% CI) | OR (95% CI) | OR (95% CI) | OR (95% CI) | OR (95% CI) | OR (95% CI) |
| Treg (%) | 1.90 (1.05 – 3.44)* | 1.88 (1.02 – 3.46)* | 1.80 (0.96 – 3.34) | 1.86 (0.97 – 3.41) | 1.67 (0.88 – 3.18) | 1.67 (0.87 – 3.19) |
| BMI (kg/m²) | 1.34 (1.01 – 1.78)* | 1.30 (0.94 – 1.81) | 1.29 (0.92 – 1.80) | 1.27 (0.89 – 1.82) | 1.20 (0.81 – 1.76) | 1.25 (0.83 – 1.87) |
| HOMA-IR | - | 1.28 (0.21 – 7.88) | 1.26 (0.14 – 8.24) | 1.13 (0.14 – 9.40) | 1.22 (0.14 – 10.43) | 1.33 (0.15 – 11.83) |
| Age | - | - | 1.11 (0.88 – 1.39) | 1.10 (0.87 – 1.39) | 1.10 (0.86 – 1.41) | 1.13 (0.87 – 1.45) |
| LDL/HDL ratio | – | - | - | 1.19 (0.26 – 5.41) | 1.33 (0.28 – 6.26) | 1.24 (0.26 – 5.98) |
| METs (min/week) | – | – | - | - | 0.51 (0.12 – 2.11) | 0.60 (0.11 – 3.35) |
| Systolic BP (mmHg) | – | – | – | - | - | 0.95 (0.85 – 1.08) |
| Χ² | 9.27* | 8.74* | 9.48* | 9.53 | 10.23 | 10.85 |

**Abbreviations:** CI: confidence interval; OR: odds ratio; BMI: body mass index; HOMA-IR: homeostasis model assessment of insulin resistance; MET: metabolic equivalent of task; BP: blood pressure. Treg: regulatory T cells.

**Table S10** Multivariable logistic regression models assessing the association of CD4+ effector memory T cells with subclinical atherosclerotic plaque status

|  | Model 1 | Model 2 | Model 3 | Model 4 | Model 5 | Model 6 |
| --- | --- | --- | --- | --- | --- | --- |
|  | OR (95% CI) | OR (95% CI) | OR (95% CI) | OR (95% CI) | OR (95% CI) | OR (95% CI) |
| CD4^+^ EM (%) | 0.95 (0.88 – 1.01) | 0.95 (0.88 – 1.02) | 0.95 (0.89 – 1.02) | 0.95 (0.89 – 1.03) | 0.96 (0.89 – 1.04) | 0.95 (0.88 – 1.04) |
| BMI (kg/m²) | 1.26 (0.97 – 1.65) | 1.19 (0.85 – 1.66) | 1.18 (0.84 – 1.67) | 1.19 (0.83 – 1.71) | 1.12 (0.77 – 1.65) | 1.19 (0.78 – 1.83) |
| HOMA-IR | - | 1.61 (0.31 – 8.45) | 1.59 (0.29 – 8.60) | 1.64 (0.25 – 10.73) | 1.62 (0.24 – 10.77) | 1.51 (0.22 – 10.47) |
| Age | - | - | 1.12 (0.88 – 1.42) | 1.12 (0.88 – 1.44) | 1.11 (0.86 – 1.43) | 1.14 (0.87 – 1.49) |
| LDL/HDL ratio | – | - | - | 0.94 (0.21 – 4.26) | 1.12 (0.12 – 5.12) | 1.19 (0.23 – 5.76) |
| METs (min/week) | – | – | - | - | 0.51 (0.12 – 2.11) | 0.57 (0.12 – 2.72) |
| Systolic BP (mmHg) | – | – | – | - | - | 0.94 (0.83 – 1.08) |
| Χ² | 7.03* | 6.66 | 7.57 | 7.58 | 8.75 | 9.59 |

**Abbreviations:** CI: confidence interval; OR: odds ratio; BMI: body mass index; HOMA-IR: homeostasis model assessment of insulin resistance; MET: metabolic equivalent of task; BP: blood pressure. CD4+ EM: CD4+ effector memory T cells.

**Table S11** Multivariable logistic regression models assessing the association of VCAM-1 with subclinical atherosclerotic plaque status

|  | Model 1 | Model 2 | Model 3 | Model 4 | Model 5 | Model 6 |
| --- | --- | --- | --- | --- | --- | --- |
|  | OR (95% CI) | OR (95% CI) | OR (95% CI) | OR (95% CI) | OR (95% CI) | OR (95% CI) |
| VCAM-1 (pg/ml) | 7.93 (1.39 – 45.30)* | 9.28 (1.31 – 65.94)* | 8.62 (1.20 – 61.84)* | 8.65 (1.20 – 62.35)* | 93.39 (2.04 – 64271.82)* | 173.02 (1.91 – 15644.46)* |
| BMI (kg/m²) | 1.82 (1.15 – 2.89)* | 2.02 (1.07 – 3.81)* | 1.99 (1.04 – 3.81)* | 1.98 (1.02 – 3.83)* | 2.37 (1.00 – 5.64) | 2.30 (0.97 – 5.42) |
| HOMA-IR | - | 0.55 (0.07 – 4.36) | 0.49 (0.06 – 4.26) | 0.46 (0.03 – 6.09) | 0.12 (0.02 – 6.38) | 0.11 (0.02 – 6.80) |
| Age | - | - | 1.11 (0.88 – 1.41) | 1.10 (0.85 – 1.43) | 1.14 (0.84 – 1.55) | 1.12 (0.81 – 1.55) |
| LDL/HDL ratio | – | - | - | 1.11 (0.18 – 6.92) | 6.44 (0.47 – 88.37) | 8.34 (0.51 – 136.46) |
| METs (min/week) | – | – | - | - | 0.01 (0.00 – 1.40) | 0.01 (0.00 – 1.31) |
| Systolic BP (mmHg) | – | – | – | - | - | 1.08 (0.88 – 1.33) |
| Χ² | 13.59*** | 13.23** | 13.99** | 14.00** | 22.92*** | 23.41*** |

Abbreviations: CI: confidence interval; OR: odds ratio; BMI: body mass index; HOMA-IR: homeostasis model assessment of insulin resistance; MET: metabolic equivalent of task; BP: blood pressure. VCAM-1: vascular cell adhesion molecule-1.

**Table S12** Multivariable logistic regression models assessing the association of CD4+ central memory T cells with subclinical atherosclerotic plaque status

|  | Model 1 | Model 2 | Model 3 | Model 4 | Model 5 | Model 6 |
| --- | --- | --- | --- | --- | --- | --- |
|  | OR (95% CI) | OR (95% CI) | OR (95% CI) | OR (95% CI) | OR (95% CI) | OR (95% CI) |
| CD4^+^CM (%) | 1.06 (0.98 – 1.16) | 1.06 (0.97 – 1.16) | 1.06 (0.97 – 1.16) | 1.06 (0.97 – 1.16) | 1.06 (0.97 – 1.16) | 1.06 (0.96 – 1.16) |
| BMI (kg/m²) | 1.27 (0.98 – 1.65) | 1.24 (0.90 – 1.70) | 1.23 (0.88 – 1.71) | 1.24 (0.87 – 1.76) | 1.14 (0.78 – 1.67) | 1.17 (0.78 – 1.76) |
| HOMA-IR | - | 1.22 (0.24 – 6.10) | 1.21 (0.22 – 6.54) | 1.29 (0.20 – 8.41) | 1.39 (0.19 – 10.04) | 1.44 (0.20 – 10.54) |
| Age | - | - | 1.18 (0.92 – 1.50) | 1.19 (0.92 – 1.54) | 1.19 (0.92 – 1.55) | 1.21 (0.92 – 1.59) |
| LDL/HDL ratio | – | - | - | 0.89 (0.21 – 5.84) | 1.13 (0.25 – 5.10) | 1.11 (0.24 – 5.08) |
| METs (min/week) | – | – | - | - | 0.37 (0.08 – 1.83) | 0.39 (0.08 – 2.05) |
| Systolic BP (mmHg) | – | – | – | - | - | 0.97 (0.85 – 1.11) |
| Χ² | 6.01* | 5.53 | 7.42 | 7.74 | 9.25 | 9.44 |

**Abbreviations:** CI: confidence interval; OR: odds ratio; BMI: body mass index; HOMA-IR: homeostasis model assessment of insulin resistance; MET: metabolic equivalent of task; BP: blood pressure. CD4+ CM: CD4+ central memory T cells.

**Table S13** Multivariable logistic regression models assessing the association of longitudinal changes in Treg/Teff ratio with subclinical atherosclerotic plaque progression

|  | Model 1 | Model 2 | Model 3 | Model 4 | Model 5 | Model 6 |
| --- | --- | --- | --- | --- | --- | --- |
|  | OR (95% CI) | OR (95% CI) | OR (95% CI) | OR (95% CI) | OR (95% CI) | OR (95% CI) |
| ΔTreg/Teff ratio | 0.28 (0.08 – 0.62)* | 0.28 (0.08 – 0.99)* | 0.08 (0.04 – 1.54) | 0.07 (0.01 – 2.07) | 0.05 (0.01 – 1.94) | 0.05 (0.01 – 2.01) |
| ΔVisceral fat | 1.26 (0.87 – 1.83) | 1.21 (0.80 – 1.82) | 0.92 (0.80 – 1.82) | 0.86 (0.43 – 1.74) | 0.87 (0.43 – 1.75) | 0.85 (0.41 – 1.78) |
| ΔVO_2_peak | - | 0.91 (0.62 – 1.34) | 0.75 (0.46 – 1.25) | 0.70 (0.42 – 1.19) | 0.69 (0.41 – 1.16) | 0.66 (0.36 – 1.21) |
| Age baseline | - | - | 1.67 (0.90 – 3.09) | 1.51 (0.75 – 3.05) | 1.54 (0.73 – 3.24) | 1.55 (0.73 – 3.26) |
| ΔHOMA-IR | – | - | - | 0.40 (0.05 – 3.80) | 0.37 (0.04 – 3.80) | 0.36 (0.04 – 3.62) |
| ΔLDL/HDL ratio | – | – | - | - | 0.32 (0.02 – 4.20) | 0.31 (0.02 – 4.05) |
| ΔSystolic BP | – | – | – | - | - | 0.98 (0.87 – 1.11) |
| Χ² | 12.62** | 12.86** | 17.67** | 18.07** | 18.99* | 19.04** |

**Abbreviations:** CI: confidence interval; OR: odds ratio; BMI: body mass index; HOMA-IR: homeostasis model assessment of insulin resistance; MET: metabolic equivalent of task; BP: blood pressure. Δ: change; Teff: T effector cells; Treg: regulatory T cells.

**Table S14** Multivariable logistic regression models assessing the association of longitudinal changes in CD8+ effector memory T cells with subclinical atherosclerotic plaque progression

|  | Model 1 | Model 2 | Model 3 | Model 4 | Model 5 | Model 6 |
| --- | --- | --- | --- | --- | --- | --- |
|  | OR (95% CI) | OR (95% CI) | OR (95% CI) | OR (95% CI) | OR (95% CI) | OR (95% CI) |
| ΔCD8+EM | 1.07 (1.01 – 1.16)* | 1.08 (0.99 – 1.16) | 1.08 (1.01 – 1.17)* | 1.07 (0.99 – 1.16) | 1.14 (1.00 – 1.30)* | 1.20 (1.00 – 1.46)* |
| ΔVisceral fat | 1.39 (1.01 – 1.95)* | 1.22 (0.85 – 1.75) | 1.11 (0.73 – 1.69) | 1.21 (0.73 – 2.00) | 1.10 (0.61 – 1.89) | 0.93 (0.48 – 1.80) |
| ΔVO_2_peak | - | 0.76 (0.52 – 1.12) | 0.69 (0.41 – 1.14) | 0.71 (0.41 – 1.21) | 0.52 (0.20 – 1.36) | 0.32 (0.06 – 1.66) |
| Age baseline | - | - | 1.23 (0.91 – 1.66) | 1.10 (0.75 – 1.62) | 1.23 (0.71 – 2.12) | 1.51 (0.73 – 2.89) |
| ΔHOMA-IR | – | - | - | 0.42 (0.02 – 7.86) | 0.21 (0.00 – 45.24) | 0.57 (0.03 – 12.02) |
| ΔLDL/HDL ratio | – | – | - | - | 0.06 (0.00 – 1.27) | 0.02 (0.00 – 1.49) |
| ΔSystolic BP | – | – | – | - | - | 0.90 (0.76 – 1.06) |
| Χ² | 13.69** | 16.65*** | 18.39*** | 18.87** | 23.88*** | 26.25*** |

**Abbreviations:** CI: confidence interval; OR: odds ratio; BMI: body mass index; HOMA-IR: homeostasis model assessment of insulin resistance; MET: metabolic equivalent of task; BP: blood pressure. ΔCD8+ EM: change in CD8+ effector memory T cells.

**Table S15** Multivariable logistic regression models assessing the association of longitudinal changes in regulatory T cells with subclinical atherosclerotic plaque progression

|  | Model 1 | Model 2 | Model 3 | Model 4 | Model 5 | Model 6 |
| --- | --- | --- | --- | --- | --- | --- |
|  | OR (95% CI) | OR (95% CI) | OR (95% CI) | OR (95% CI) | OR (95% CI) | OR (95% CI) |
| ΔTreg | 0.74 (0.42 – 1.29) | 0.60 (0.32 – 1.25) | 0.53 (0.32 – 1.17) | 0.46 (0.17 – 1.24) | 0.36 (0.11 – 1.23) | 0.34 (0.08 – 1.39) |
| ΔVisceral fat | 1.41 (0.98 – 2.03) | 1.23 (0.82 – 1.84) | 1.15 (0.73 – 1.79) | 1.20 (0.70 – 2.05) | 1.19 (0.68 – 2.06) | 1.16 (0.65 – 2.10) |
| ΔVO_2_peak | - | 0.76 (0.56 – 1.03) | 0.70 (0.49 – 1.01) | 0.74 (0.50 – 1.08) | 0.72 (0.48 – 1.09) | 0.64 (0.36 – 1.13) |
| Age baseline | - | - | 1.24 (0.90 – 1.71) | 1.00 (0.67 – 1.51) | 0.98 (0.63 – 1.54) | 1.03 (0.65 – 1.64) |
| ΔHOMA-IR | – | - | - | 0.15 (0.01 – 3.04) | 0.08 (0.01 – 3.37) | 0.09 (0.01 – 4.49) |
| ΔLDL/HDL ratio | – | – | - | - | 0.17 (0.01 – 2.84) | 0.18 (0.01 – 2.66) |
| ΔSystolic BP | – | – | – | - | - | 0.96 (0.86 – 1.07) |
| Χ² | 10.08** | 13.87** | 15.65** | 18.56** | 20.90** | 21.57** |

**Abbreviations:** CI: confidence interval; OR: odds ratio; BMI: body mass index; HOMA-IR: homeostasis model assessment of insulin resistance; MET: metabolic equivalent of task; BP: blood pressure. ΔTreg: change in regulatory T cells.

**Table S16** Multivariable logistic regression models assessing the association of longitudinal changes in CD4+ central memory T cells with subclinical atherosclerotic plaque progression

|  | Model 1 | Model 2 | Model 3 | Model 4 | Model 5 | Model 6 |
| --- | --- | --- | --- | --- | --- | --- |
|  | OR (95% CI) | OR (95% CI) | OR (95% CI) | OR (95% CI) | OR (95% CI) | OR (95% CI) |
| ΔCD4CM | 0.93 (0.84 – 1.02) | 0.93 (0.84 – 1.03) | 0.88 (0.76 – 1.12) | 0.86 (0.72 – 1.03) | 0.87 (0.71 – 1.07) | 0.88 (0.71 – 1.07) |
| ΔVisceral fat | 1.51 (1.05 – 2.18)* | 1.39 (0.94 – 2.05) | 1.32 (0.86 – 2.02) | 1.56 (0.84 – 2.91) | 1.55 (0.83 – 2.87) | 1.47 (0.79 – 2.73) |
| ΔVO_2_peak | - | 0.84 (0.63 – 1.12) | 0.78 (0.56 – 1.08) | 0.85 (0.58 – 1.25) | 0.85 (0.57 – 1.25) | 0.77 (0.49 – 1.23) |
| Age baseline | - | - | 1.33 (0.96 – 1.86) | 1.12 (0.77 – 1.64) | 1.11 (0.76 – 1.64) | 1.13 (0.77 – 1.65) |
| ΔHOMA-IR | – | - | - | 0.19 (0.01 – 3.82) | 0.20 (0.01 – 4.11) | 0.25 (0.02 – 3.69) |
| ΔLDL/HDL ratio | – | – | - | - | 0.76 (0.07 – 8.69) | 0.68 (0.06 – 7.72) |
| ΔSystolic BP | – | – | – | - | - | 0.95 (0.85 – 1.07) |
| Χ² | 11.43** | 13.15** | 16.35** | 18.90** | 18.95** | 19.73** |

**Abbreviations:** CI: confidence interval; OR: odds ratio; BMI: body mass index; HOMA-IR: homeostasis model assessment of insulin resistance; MET: metabolic equivalent of task; BP: blood pressure. ΔCD4+ CM: change in CD4+ central memory T cells.

**Table S17** Multivariable logistic regression models assessing the association of longitudinal changes in CD8+ EMRA T cells with subclinical atherosclerotic plaque progression

|  | Model 1 | Model 2 | Model 3 | Model 4 | Model 5 | Model 6 |
| --- | --- | --- | --- | --- | --- | --- |
|  | OR (95% CI) | OR (95% CI) | OR (95% CI) | OR (95% CI) | OR (95% CI) | OR (95% CI) |
| ΔCD8EMRA | 0.95 (0.88 – 1.03) | 0.96 (0.89 – 1.03) | 0.96 (0.89 – 1.03) | 0.95 (0.87 – 1.05) | 0.96 (0.88 – 1.05) | 0.96 (0.88 – 1.05) |
| ΔVisceral fat | 1.36 (0.97 – 1.91) | 1.24 (0.85 – 1.79) | 1.18 (0.79 – 1.77) | 1.29 (0.79 – 2.09) | 1.31 (0.79 – 2.17) | 1.27 (0.77 – 2.11) |
| ΔVO_2_peak | - | 0.79 (0.55 – 1.14) | 0.76 (0.51 – 1.13) | 0.71 (0.42 – 1.20) | 0.72 (0.42 – 1.21) | 0.67 (0.38 – 1.17) |
| Age baseline | - | - | 1.14 (0.89 – 1.47) | 0.97 (0.68 – 1.37) | 0.98 (0.68 – 1.41) | 1.02 (0.71 – 1.47) |
| ΔHOMA-IR | – | - | - | 0.14 (0.01 – 4.76) | 0.14 (0.01 – 5.50) | 0.22 (0.01 – 6.19) |
| ΔLDL/HDL ratio | – | – | - | - | 0.37 (0.04 – 3.14) | 0.35 (0.04 – 3.05) |
| ΔSystolic BP | – | – | – | - | - | 0.95 (0.86 – 1.05) |
| Χ² | 11.03** | 13.24** | 14.28** | 16.92** | 17.89** | 18.90** |

**Abbreviations:** CI: confidence interval; OR: odds ratio; BMI: body mass index; HOMA-IR: homeostasis model assessment of insulin resistance; MET: metabolic equivalent of task; BP: blood pressure. ΔCD8+ EMRA: change in CD8+ EMRA T cells.

**Table S18** Multivariable logistic regression models assessing the association of longitudinal changes in CXCL-9 with subclinical atherosclerotic plaque progression

|  | Model 1 | Model 2 | Model 3 | Model 4 | Model 5 | Model 6 |
| --- | --- | --- | --- | --- | --- | --- |
|  | OR (95% CI) | OR (95% CI) | OR (95% CI) | OR (95% CI) | OR (95% CI) | OR (95% CI) |
| ΔCXCL9 | 2.15 (0.67 – 6.89) | 1.89 (0.53 – 6.72) | 1.73 (0.47 – 6.33) | 1.62 (0.40 – 6.56) | 1.30 (0.30 – 5.61) | 1.23 (0.27 – 5.51) |
| ΔVisceral fat | 1.47 (1.04 – 2.08)* | 1.36 (0.93 – 1.99) | 1.33 (0.90 – 1.98) | 1.45 (0.90 – 2.32) | 1.47 (0.90 – 2.42) | 1.46 (0.89 – 2.40) |
| ΔVO_2_peak | - | 0.84 (0.57 – 1.22) | 0.81 (0.55 – 1.20) | 0.77 (0.48 – 1.21) | 0.77 (0.48 – 1.23) | 0.72 (0.44 – 1.18) |
| Age baseline | - | - | 1.13 (0.87 – 1.47) | 0.97 (0.67 – 1.38) | 0.98 (0.67 – 1.43) | 1.01 (0.69 – 1.48) |
| ΔHOMA-IR | – | - | - | 0.15 (0.01 – 4.23) | 0.14 (0.01 – 4.81) | 0.21 (0.01 – 5.62) |
| ΔLDL/HDL ratio | – | – | - | - | 0.36 (0.05 – 2.73) | 0.33 (0.04 – 2.64) |
| ΔSystolic BP | – | – | – | - | - | 0.95 (0.87 – 1.05) |
| Χ² | 11.29** | 12.28** | 13.12** | 15.77** | 16.92** | 17.88* |

**Abbreviations:** CI: confidence interval; OR: odds ratio; BMI: body mass index; HOMA-IR: homeostasis model assessment of insulin resistance; MET: metabolic equivalent of task; BP: blood pressure. CXCL-9: chemokine (C-X-C motif) ligand 9.

**Table S19** Multivariable logistic regression models assessing the association of longitudinal changes in memory regulatory T cells with subclinical atherosclerotic plaque progression

|  | Model 1 | Model 2 | Model 3 | Model 4 | Model 5 | Model 6 |
| --- | --- | --- | --- | --- | --- | --- |
|  | OR (95% CI) | OR (95% CI) | OR (95% CI) | OR (95% CI) | OR (95% CI) | OR (95% CI) |
| ΔmTreg | 0.98 (0.94 – 1.02) | 0.98 (0.95 – 1.02) | 0.98 (0.93 – 1.02) | 0.97 (0.93 – 1.02) | 0.98 (0.93 – 1.03) | 0.98 (0.93 – 1.04) |
| ΔVisceral fat | 1.50 (1.05 – 2.14)* | 1.38 (0.94 – 2.12) | 1.33 (0.88 – 2.00) | 1.48 (0.88 – 2.47) | 1.48 (0.88 – 2.47) | 1.46 (0.87 – 2.45) |
| ΔVO_2_peak | - | 0.84 (0.61 – 1.15) | 0.82 (0.58 – 1.15) | 0.80 (0.50 – 1.27) | 0.80 (0.50 – 1.25) | 0.74 (0.46 – 1.21) |
| Age baseline | - | - | 1.24 (0.91 – 1.68) | 1.04 (0.72 – 1.50) | 1.02 (0.70 – 1.49) | 1.05 (0.72 – 1.53) |
| ΔHOMA-IR | – | - | - | 0.14 (0.01 – 3.17) | 0.13 (0.01 – 3.83) | 0.18 (0.01 – 4.62) |
| ΔLDL/HDL ratio | – | – | - | - | 0.50 (0.05 – 4.60) | 0.46 (0.04 – 4.74) |
| ΔSystolic BP | – | – | – | - | - | 0.96 (0.87 – 1.05) |
| Χ² | 10.21** | 11.72** | 13.70** | 16.90** | 17.31** | 18.18* |

**Abbreviations:** CI: confidence interval; OR: odds ratio; BMI: body mass index; HOMA-IR: homeostasis model assessment of insulin resistance; MET: metabolic equivalent of task; BP: blood pressure. mTreg: memory regulatory T cells.
